# ELLIPSIS: robust quantification of splicing in scRNA-seq

**DOI:** 10.1093/bioinformatics/btaf028

**Published:** 2025-02-12

**Authors:** Marie Van Hecke, Niko Beerenwinkel, Thibault Lootens, Jan Fostier, Robrecht Raedt, Kathleen Marchal

**Affiliations:** IDLab, Department of Information Technology, Ghent University-IMEC, 9052 Ghent, Belgium; Department of Plant Biotechnology and Bioinformatics, Ghent University, 9052 Ghent, Belgium; Cancer Research Institute Ghent (CRIG), Ghent University, 9000 Ghent, Belgium; Department of Biosystems Science and Engineering, ETH Zürich, 4056 Basel, Switzerland; SIB Swiss Institute of Bioinformatics, 4051 Basel, Switzerland; Cancer Research Institute Ghent (CRIG), Ghent University, 9000 Ghent, Belgium; 4Brain, Department of Head and Skin, Ghent University, 9000 Ghent, Belgium; Laboratory of Experimental Cancer Research, Department of Human Structure and Repair, Ghent University, 9000 Ghent, Belgium; Department of Plant Biotechnology and Bioinformatics, Ghent University, 9052 Ghent, Belgium; Cancer Research Institute Ghent (CRIG), Ghent University, 9000 Ghent, Belgium; 4Brain, Department of Head and Skin, Ghent University, 9000 Ghent, Belgium; IDLab, Department of Information Technology, Ghent University-IMEC, 9052 Ghent, Belgium; Department of Plant Biotechnology and Bioinformatics, Ghent University, 9052 Ghent, Belgium; Cancer Research Institute Ghent (CRIG), Ghent University, 9000 Ghent, Belgium

## Abstract

**Motivation:**

Alternative splicing is a tightly regulated biological process, that due to its cell type specific behavior, calls for analysis at the single cell level. However, quantifying differential splicing in scRNA-seq is challenging due to low and uneven coverage. Hereto, we developed ELLIPSIS, a tool for robust quantification of splicing in scRNA-seq that leverages locally observed read coverage with conservation of flow and intra-cell type similarity properties. Additionally, it is also able to quantify splicing in novel splicing events, which is extremely important in cancer cells where lots of novel splicing events occur.

**Results:**

Application of ELLIPSIS to simulated data proves that our method is able to robustly estimate Percent Spliced In values in simulated data, and allows to reliably detect differential splicing between cell types. Using ELLIPSIS on glioblastoma scRNA-seq data, we identified genes that are differentially spliced between cancer cells in the tumor core and infiltrating cancer cells found in peripheral tissue. These genes showed to play a role in a.o. cell migration and motility, cell projection organization, and neuron projection guidance.

**Availability and implementation:**

ELLIPSIS quantification tool: https://github.com/MarchalLab/ELLIPSIS.git.

## 1 Introduction

In the intricate realm of molecular biology, RNA splicing, the process by which intronic regions are excised and exonic regions are joined together, plays a pivotal role in generating distinct mRNA transcripts from a single gene, governing the diversity and functionality of the transcriptome. Alternatively spliced transcripts of the same gene can exhibit vastly different or even opposite functions within the cell (Bowler and Oltean 2019), which is neglected in traditional gene expression analysis. Additionally, bulk RNA analysis suffers from the unknown cell type composition, which is a confounding factor that is hard to account for in highly cell type specific processes such as splicing. Advances in single cell sequencing technologies (Picelli *et al.* 2014, Hagemann-Jensen *et al.* 2020, 2022) enable alternative splicing analysis at single cell resolution, providing insights into cellular heterogeneity, developmental processes, and disease mechanisms by capturing how splicing dynamics contribute to cellular identity, response to stimuli, and disease progression. However, identifying and quantifying differential splicing from single cell data remain complex, due to low and uneven coverage across transcripts resulting from the low capture rate and high number of PCR amplification cycles. Additionally, several distinct differentially spliced transcripts might be present within a single cell, exhibiting extensive combinations of common splice variants (Supplementary Fig. S1).

Most techniques for the quantification of splicing in bulk RNA-seq data (Katz *et al.* 2010, Trapnell *et al.* 2010, Li *et al.* 2018, Vaquero-Garcia *et al.* 2023) rely on constructing splice graphs and mapping reads to those splice graphs in order to estimate inclusion ratios, or Percent Spliced In (Ψ) values. However, single-cell RNA-seq data suffer from low and uneven coverage, which cannot be dealt with by tools developed for bulk RNA-seq data. Therefore, several single cell splicing analysis tools (Huang and Sanguinetti 2021, Gilis *et al.* 2021, Buen Abad Najar *et al.* 2022) have been developed. In contrast to bulk tools, none of the existing single cell tools allow for the detection and quantification of novel splicing events, which is important in cancer where lots of novel splicing events might occur (Calabrese *et al.* 2020, Karakulak *et al.* 2021). Additionally, most tools (Huang and Sanguinetti 2021, Buen Abad Najar *et al.* 2022) do not allow for the quantification of complex splicing events, and only detect differentially spliced cassette exons (Supplementary Fig. S1A). Finally, even when they are developed for single cell data, most tools fail to reliably detect differential splicing in cells with low read coverage, making robust estimation of Ψ-values a persistent challenge.

**Figure 1.**
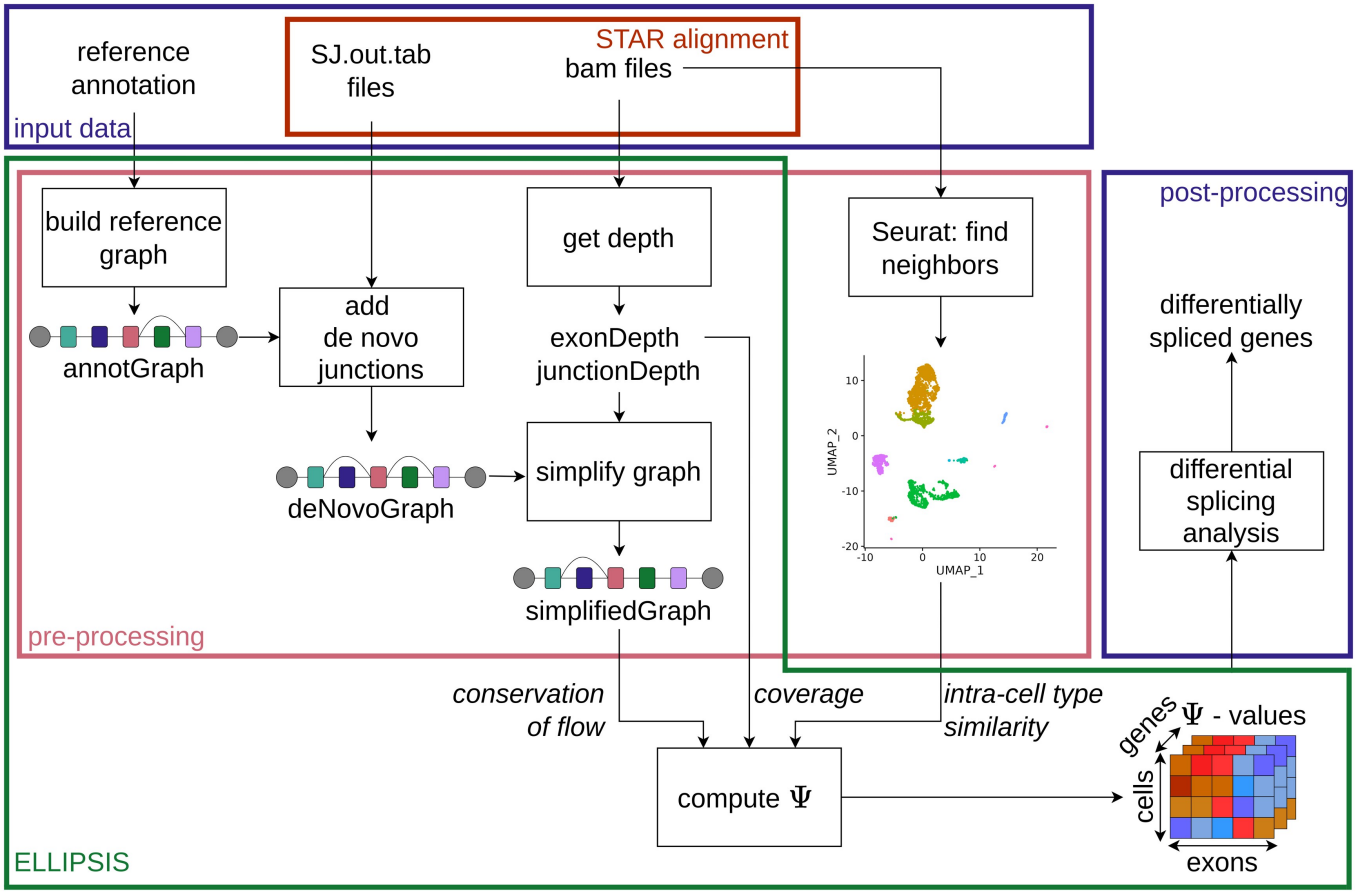
Overview of the different inputs, outputs, and analysis steps required for differential splicing detection. Given the reference annotation and the STAR alignment results, ELLIPSIS builds a splice graph for each gene using three consecutive steps: building reference graphs, enriching the graphs with *de novo* splicing events, and simplifying the graphs by removing unobserved exons and junctions. Similar cells are identified with Seurat findNeighbors. Next, ELLIPSIS computes Ψ-values, which can then be analysed further in post-processing to identify differentially spliced genes.

To cope with the aforementioned drawbacks of current methods, we here propose ELLIPSIS, a graph-based method that leverages intra-cell type similarity and conservation of flow properties for robust splicing quantification from Smart-seq data, see overview in Fig. 1. The scarcity of single cell data leads to low and uneven coverage across transcripts, resulting in inconsistent Ψ-values when solely relying on read coverage. Therefore, we leverage the locally observed read coverage with information obtained from conservation of flow and intra-cell type similarity. The conservation of flow ensures that Ψ-values are consistent throughout splice graphs by maintaining a local balance at each exon: the sum of Ψ-values of the incoming junctions has to be equal to the Ψ-value of the exon itself, and the same holds for the outgoing junctions; similarly to conservation of flow of multiplicities described in (Steyaert *et al.* 2020). Because cells from the same cell type tend to exhibit the same splice patterns, we use intra-cell type similarity to enrich the read coverage from one cell with the read information from similar cells, improving Ψ-estimates for cells with low gene coverage. For each cell, we use the subset of cells with high gene expression similarity to allow assessing splice variation between distinct cell types and during continuous biological processes, e.g. along a trajectory of developing cells.

## 2 Materials and methods

### 2.1 Building splice graphs

To quantify both known and novel splicing events, we first construct reference-based splice graphs for each gene by creating nodes for each exon in the annotation, and connecting nodes that occur consecutively in the annotated transcripts. We also include an artificial source and sink node, which are respectively connected to the first/last exons of each transcript in the reference annotation. Subsequently, the splice graphs are extended by adding novel junctions observed in the read data, accepting only those that are supported by at least five reads in at least 10 cells to avoid spurious junctions. This addition of novel junctions enables the detection and quantification of novel splice variants that have not yet been annotated. Finally, the graphs are simplified by removing exons and junctions that have a read coverage <1 in all cells, unless they are required for graph connectivity.

**Figure 2.**
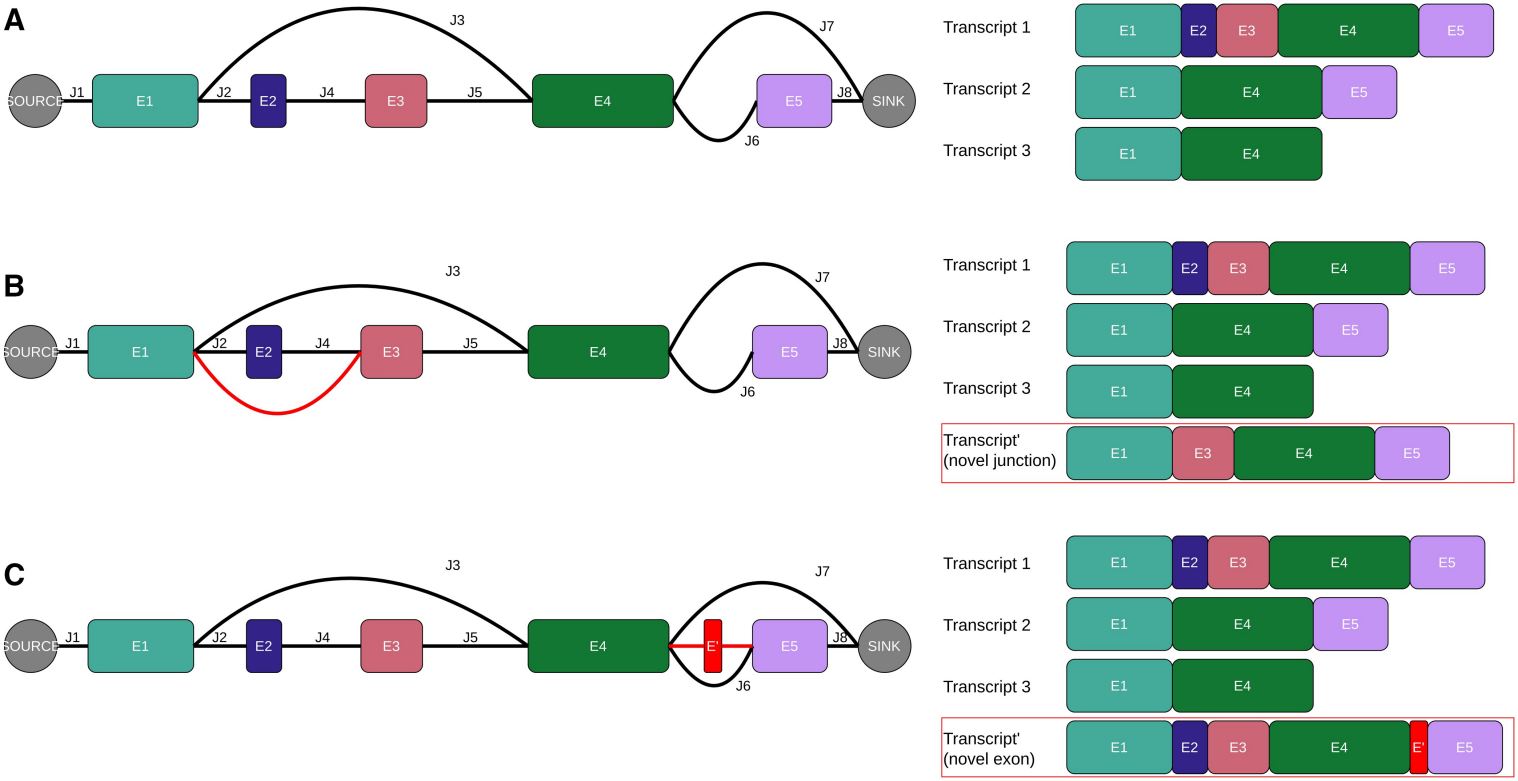
Splice graphs for a gene with three annotated transcripts and different *de novo* splicing events. (A) Splice graph corresponding with three annotated transcripts. (B) Splice graph for the same three annotated transcripts and an additional transcript containing a novel junction between E1 and E2 (red). (C) Splice graph for three annotated transcripts and an additional transcript containing a novel exon E' (red). This novel exon is connected to the graph by two additional novel junctions.

As artificial edges connected to the source/sink nodes cannot be observed in the reads, it is impossible to add novel connections based on observed junction spanning counts. Therefore, the quantification of first/last exons is limited to those present in the reference annotation. An example of alternative last exon usage can be found in Fig. 2A: E4 and E5 can both be a last exon, which is why they are connected to the sink node by junctions J7 and J8, respectively.

### 2.2 Estimation of Ψ-values

To quantify alternative splicing, we compute Ψ-values for each exon and junction in the splice graph for every cell, representing the fraction of transcripts in the cell that contain that specific exon/junction. To obtain robust estimates of these Ψ-values, we use three types of information: observed read coverage, conservation of flow, and intra-cell type similarity.

The observed read coverage of an exon/junction is proportional to its Ψ-value, but is affected by read errors, mapping issues, GC-bias, and technical biases, resulting in uneven coverage. To address this, we use the property of conservation of flow. Because each transcript of a gene can be represented as a path from the source to the sink in the graph, and all transcripts come together at the source and sink node; these nodes have Ψ=100%. Additionally, transcripts cannot start or end in other nodes, which means we can define the conservation of flow property locally for every node as: the sum of incoming Ψ is equal to Ψ of the node itself, and to the sum of the outgoing Ψ.

In addition, estimation of Ψ-values is complicated by the low coverage inherent to single cell data. To address these challenges, we assume that cells from the same cell type have similar gene expression and transcript usage. Hereto, we us intra-cell type similarity to impose that cells with a similar gene expression pattern also have similar Ψ-values. This property leverages high-quality information from cells with high coverage to improve the Ψ-estimates for similar cells with low coverage, effectively addressing the challenges posed by low coverage in single-cell RNA-seq data.

Finally, we account for differences in gene expression and sequencing depth between cells by introducing *α^c^*, a proxy for the cell-specific gene coverage, representing the expected coverage for an exon/junction with Ψ=100% in cell *c*.

#### 2.2.1 Constraints

We represent each of the three types of information with specific equations, forming an overdetermined system that is optimized using weighted least squares. First, the observed coverage $a_{i}^{c}$ of exon/junction *i* in cell *c* is proportional to the corresponding $\Psi_{i}^{c}$
 

(1) $$\Psi_{i}^{c}=\frac{a_{i}^{c}}{\alpha^{c}}\;\forall i\in \tilde{E}\cup \tilde{J}$$

We obtain one equation for each observable exon $e\in \tilde{E}$ and junction $j\in \tilde{J}$, i.e. all exons, except the source and sink, and all junctions except those directly connected to the source/sink.

Next, we represent the conservation of flow of Ψ-values in each exon *e*. In cell *c*, the Ψ-value of exon *e* itself, $\Psi_{e}^{c}$, is equal to the sum of the Ψ-values of *e*’s incoming junctions j∈In(e), and similarly for the outgoing junctions j∈Out(e)
 

(2) $$\begin{array}{cc} \Psi_{e}^{c}=\underset{j\in \text{In}(e)}{\sum }\Psi_{j}^{c}\; & \forall e\in E\setminus \{\text{source}\}\\ \Psi_{e}^{c}=\underset{j\in \text{Out}(e)}{\sum }\Psi_{j}^{c}\; & \forall e\in E\setminus \{\text{sink}\} \end{array}$$

There are two equations for each exon in the graph, except for the source and sink node where the equation for the incoming, resp. outgoing junctions is replaced with:

(3) $$\begin{array}{c} \Psi_{\text{source}}^{c}=100\%\\ \Psi_{\text{sink}}^{c}=100\% \end{array}$$

Conservation of flow accounts for local coverage bias, but also allows for the quantification of unobservable junctions, i.e. the junctions connected to source/sink. Even though we cannot estimate their Ψ-values from observed reads, we can infer them using the Ψ-values of the exons they connect. Those exons are observable, or known to have Ψ=100% (source and sink nodes). This allows the analysis of differential first/last exon usage.

Finally, we represent intra-cell type similarity by calculating a weighted average of the Ψ-values from cells that are similar to cell *c*. Hereto, we consider all cells *d* with gene expression patterns similar to cell *c*, denoted as d∈N(c). We use *α^d^* as weights, because cells with a higher coverage typically suffer less from uneven coverage, and therefore have more reliable Ψ-values

(4) $$\Psi_{i}^{c}=\frac{\sum_{d\in N(c)}\alpha^{d}\Psi_{i}^{d}}{\sum_{d\in N(c)}\alpha^{d}}\;\forall i\in E\cup J$$

This equation is defined for all exons *E* and junctions *J*.

The combined set of constraints (1)–(4), results in an overdetermined system (6). For each cell *c*, we have a value *α^c^*, as well as a $\Psi_{i}^{c}$-value for each exon/junction *i*, resulting in a large number of variables and a complex minimization problem. Therefore, we use an iterative approach similar to the expectation–maximization algorithm. In the expectation step, we consider *α^c^* fixed and optimize the Ψ-values to minimize the sum of squared residuals. In the maximization step, we keep the Ψ-values fixed, and compute *α^c^* for each cell using the observed coverage. Because both the number of equations and the number of variables scale linearly with the number of cells, we compute the expectation and maximization step for each cell separately and in parallel in each iteration to maintain scalability. Hereto, we adapt Eq. (4) such that we use the Ψ′-values from the previous EM-iteration

(5) $$\Psi_{i}^{c}=\frac{\sum_{d\in N(c)}\alpha^{d}\Psi \prime_{i}^{d}}{\sum_{d\in N(c)}\alpha^{d}}\;\forall i\in E\cup J$$

#### 2.2.2 Expectation

Combining all constraints described above leads to an overdetermined system, which we solve using weighted least squares (WLS). We assign a weight to each type of equation, relative to the weight for the observed coverage equations (1), which is fixed to 1. We include following weights: $w_{\text{flow}}$ for the conservation of flow equations (2), $w_{\text{ss}}$ for the source/sink equations (3), and $w_{\text{sim}}$ for the intra-cell type similarity equations (4). We solve the following set of equations using WLS:

(6) $$\{\begin{array}{cc} \Psi_{i}^{c}=\frac{a_{i}^{c}}{\alpha^{c}} & \forall i\in \tilde{E}\cup \tilde{J}\\ w_{\text{flow}}(\Psi_{e}^{c}-\underset{j\in \text{In}(e)}{\sum }\Psi_{j}^{c})=0 & \forall e\in E\setminus \text{source}\\ w_{\text{flow}}(\Psi_{e}^{c}-\underset{j\in \text{Out}(e)}{\sum }\Psi_{j}^{c})=0 & \forall e\in E\setminus \text{sink}\\ w_{\text{ss}}\Psi_{\text{source}}^{c}=w_{\text{ss}} & \\ w_{\text{ss}}\Psi_{\text{sink}}^{c}=w_{\text{ss}} & \\ w_{\text{sim}}\Psi_{i}^{c}=w_{\text{sim}}\frac{\sum_{d\in N(c)}\alpha^{d}\Psi_{i}\prime^{d}}{\sum_{d\in N(c)}\alpha^{d}} & \forall i\in E\cup J \end{array}$$

where each equation *n* can be written as an equation with a linear combination, $f_{n}(\Psi_{i}^{c})$, of unknown Ψ-values on the left-hand side, and a known value *y_n_* on the right-hand side:

(7) $$f_{n}(\Psi_{i}^{c})=y_{n}$$

We find the optimal values for $\Psi_{i}^{c}$ for all exons/junctions *i*, by minimizing the sum of squared residuals:

(8) $$\text{min}\sum_{n}{\left(y_{n}-f_{n}(\Psi_{i}^{c})\right)}^{2}$$

An extensive grid search on simulated data determined the optimal weights: $w_{\text{flow}}=6,\;w_{\text{ss}}=1$ and $w_{\text{sim}}=4$ (Supplementary Material S7).

#### 2.2.3 Intermediate step

**Figure 3.**
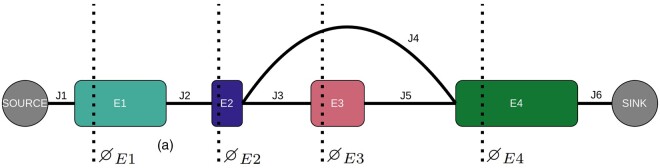
Splice graph with cross sections for each observable exon. Cross sections $\emptyset_{E1},\;\emptyset_{E2}$ and $\emptyset_{E4}$ only contain their own exon, while cross section $\emptyset_{E3}$ also contains junction J4.

We insert an additional step between the expectation and maximization step to prevent small errors from propagating. If, in an early iteration, most computed Ψ-values are too low, the maximization step will result in an estimate for *α* that is too high. Since Ψ-values depend on the ratio of observed coverage to *α*  (1), this will result in even lower Ψ-values in subsequent iterations. The local constraint of conservation of flow may not be strong enough to counter this effect, leading to an EM algorithm that never converges. To avoid this issue, we impose global conservation of flow to ensure the sum of Ψ-values in each cross section of the splice graph equals 100%. A cross section $\emptyset_{e}$ of the splice graph at exon *e* is defined as the set containing exon *e* itself, and all the junctions that start before and end after exon *e*, see Fig. 3.

In the Intermediate step we rescale the Ψ′-values from the Expectation step, such that the average Ψ-value of all cross sections ${\bar{\Psi }}_{\emptyset }$ equals 100%

(9) $$\begin{array}{l} {\bar{\Psi \prime }}_{\emptyset }^{c}=\frac{\sum_{e\in E}\sum_{i\in \emptyset_{e}}\Psi \prime_{i}^{c}}{\left|E\right|}\\ \Psi_{i}^{c}=\frac{\Psi \prime_{i}^{c}}{{\bar{\Psi \prime }}_{\emptyset }^{c}}\;\forall i\in E\cup J \end{array}$$

#### 2.2.4 Maximization

In the maximization step, we compute the gene coverage *α^c^* of cell *c*, using the Ψ-values from the intermediate step, and the observed coverage $a_{i}^{c}$ of all observable exons and junctions *i*. Because some junctions are harder to map due to small read overhangs, we use a different value for the exons ($\alpha_{E}^{c}$) and the junctions ($\alpha_{J}^{c}$)

(10) $$\begin{array}{cc} \sqrt{l_{e}}\;\Psi_{e}^{c}\;\alpha_{E}^{c}=\sqrt{l_{e}}\;a_{e}^{c} & \;\forall e\in \tilde{E}\\ \Psi_{j}^{c}\;\alpha_{J}^{c}=a_{j}^{c} & \;\forall j\in \tilde{J} \end{array}$$

For exons, we use weights proportional to their length *l_e_*, because the observed exon coverage is averaged over all bases, which smooths local coverage variations in longer exons, providing more reliable coverage. We solve these equations using a WLS approach to determine $\alpha_{E}^{c}$ and $\alpha_{J}^{c}$:

(11) $$\text{min}\left(\sum_{e\in \tilde{E}}l_{e}{\left(a_{e}^{c}-\Psi_{e}^{c}\;\alpha_{E}^{c}\right)}^{2}+\sum_{j\in \tilde{J}}{\left(a_{j}^{c}-\Psi_{j}^{c}\;\alpha_{J}^{c}\right)}^{2}\right)$$

#### 2.2.5 Initialization

*α^c^* is initialized using the exons with highest coverage per cell *c*. We select the *n* exons with highest coverage, until their combined lengths are at least 10% of the length of the longest annotated transcript in the graph. Using multiple exons prevents an extremely high initial value of *α^c^*, due to a small exon with very high coverage. We use the average coverage of these selected exons as the initial value for *α_E_* and *α_J_*.

#### 2.2.6 Convergence criterion

We consider the EM algorithm to be converged if the difference between the Ψ-values computed in the previous iteration and the current iteration is smaller than 0.01% for all exons and junctions in each cell.

### 2.3 Filtering genes and cells

We apply the methodology described above to obtain Ψ-values for all genes in the genome and all cells in the dataset. To reduce unnecessary compute time, Ψ-values for certain genes and cells are not estimated. During the simplify graph step, we filter out genes that do not have at least one cell with coverage >10, as they lack sufficient coverage for accurate Ψ-value estimation. Additionally, splice graphs that are entirely sequential are removed, as they do not allow for alternative splicing.

Further filtering is applied before the Ψ-values are computed. Firstly, we exclude extremely complex graphs with more than 1 million possible paths from source to sink, to save memory, and avoid inaccurate Ψ-estimates. Additionally, the Ψ-values are not computed for cells where over 10% of mapped reads for a gene have a low-quality score (MAPQ < 10), as this indicates mapping difficulties, which could result in unreliable Ψ-estimates. Finally, cells with low gene expression ($\alpha^{c}<10$), do not have enough read coverage to accurately determine Ψ-values, and are therefore removed.

### 2.4 Bioinformatics methods

Identification of differentially expressed genes is performed using the *findMarkers*-function, while cells with similar gene expression are found by using the *findNeighbors*-function from Seurat (Hao *et al.* 2024). Additionally, gene fusion detection is performed using scFusion (Jin *et al.* 2022).

For the analysis of splice regulators, we used SpliceAid (Giulietti *et al.* 2013), a database containing experimentally validated RNA-splice factor interactions for 71 splice factors; and the clipDB database (Zhao *et al.* 2022), containing couples of 221 RNA-binding proteins (RBPs) and their target genes. For the clipDB database, we only selected the 62 RBPs that are known to have a splicing regulation function, or are part of the spliceosome. To identify overrepresentation of splice interaction targets, we use a hyper-geometric test and Benjamini–Hochberg multiple hypothesis correction.

For benchmarking against state-of-the-art single cell splicing analysis tools, we applied Psix (Buen Abad Najar *et al.* 2022), BRIE2 (Huang and Sanguinetti 2021), and satuRn (Gilis *et al.* 2021) to the same data as ELLIPSIS. Psix requires a latent space to detect similar cells using their own distance metrics. Hereto, we used the PCA coordinates obtained by applying the standard Seurat pipeline (Hao *et al.* 2024) to raw gene counts, which consists of normalization, scaling, and PCA reduction. We also used parameter *n_neighbors *=* *20 while running Psix.

For the analysis with BRIE2, we used the most recent human splice event annotation available on sourceforge (gencode.v27), and ran *brie-count* with default parameters, followed by *brie-quant* in mode2 to find differential splicing between two cell types, with *–interceptMode gene*.

Before running satuRn with default parameters, we performed transcriptome alignment with *salmon-quant* (Patro *et al.* 2017) in alignment-based mode using default arguments.

## 3 Results

### 3.1 Performance assessment on simulated data

To assess the performance of ELLIPSIS, we simulated splice-aware reads that mimic data generated by Smart-seq 2 (Picelli *et al.* 2014). In contrast to previously published simulators that generate only gene expression counts or splice junction counts (Zhang *et al.* 2019, Sun *et al.* 2021), we require both splice junction counts and exon-level coverage information. Hereto, we implemented a simulation pipeline that generates raw reads in fastq format, which are then aligned to the reference genome, ensuring any mapping bias introduced during alignment is inherently included in the simulation.

We mimic a situation with two cell types, each consisting of 100 homogeneous cells. These cells each express 100 genes, with 50 genes being differentially spliced and 50 not. Within a cell type, the gene expression only differs by cell intrinsic factors, such as the cell size. In contrast, cells from different cell types can also differ in splice patterns, meaning alternatively spliced transcripts are present in different proportions. For more details about the setup of this simulation, see Supplementary Material S3.

We create four different datasets: each consisting of three annotated transcripts, supplemented with 0, 1, or 2 additional transcripts with novel splicing events. The first dataset (allAnnot) only contains three annotated transcripts per gene, the second dataset (novelJ) contains one additional transcript with an unannotated junction per gene, the third dataset (novelE) contains an additional transcript with an unannotated exon (and its two corresponding new junctions) per gene, and the fourth dataset (novelEJ) contains two additional transcripts per gene, one with a novel exon and one with a novel junction. Figure 2 shows an example of such annotated and non-annotated transcripts and their corresponding splice graphs.

Raw reads are simulated with Polyester (Frazee *et al.* 2015) and preprocessed (Supplementary Material S3). Running ELLIPSIS on this simulated data produces Ψ-values for all genes across all cells, except for gene–cell combinations with low coverage ($\alpha^{c}<10$) or a high percentage of low-quality mapped reads (over 10% low-quality reads). Genes for which none of the simulated cells pass this filter do not have any Ψ-values computed and are therefore excluded from further analysis.

#### 3.1.1 Accuracy of Ψ-values

To assess the accuracy of the predicted Ψ-values, we compared them to the ground truth Ψ-values. For each cell, we calculated the average absolute exon error per gene, defined as the mean absolute difference between the estimated and true Ψ-values across all base pairs contained in the exons of the corresponding splice graph.

To ensure that genes with numerous small exons are not disproportionately penalized, we computed the error at the base-pair level rather than the exon level. Calculating the error at the exon level could otherwise inflate the average gene error in cases where differential 3′/5′ splicing subdivides exons into multiple partial exons. As an example, we consider the splice graphs in Supplementary Fig. S3, and assume that ELLIPSIS estimates all Ψ-values correctly except for exon E3, where there is a 10% error. In splice graph A, this results in an average error of 2.5% at the exon level ($\frac{10\%\;\text{error}}{4\;\text{exons}}$). In splice graph B, where E3 is split into partial exons, the same 10% error for E3 would result in a doubled exon-level error of 5% ($\frac{3\cdot 10\%\;\text{error}}{6\;\text{exons}}$). Thus, to prevent overestimation of errors in genes composed of multiple small exons, we calculated the error at the base-pair level.

**Figure 4.**
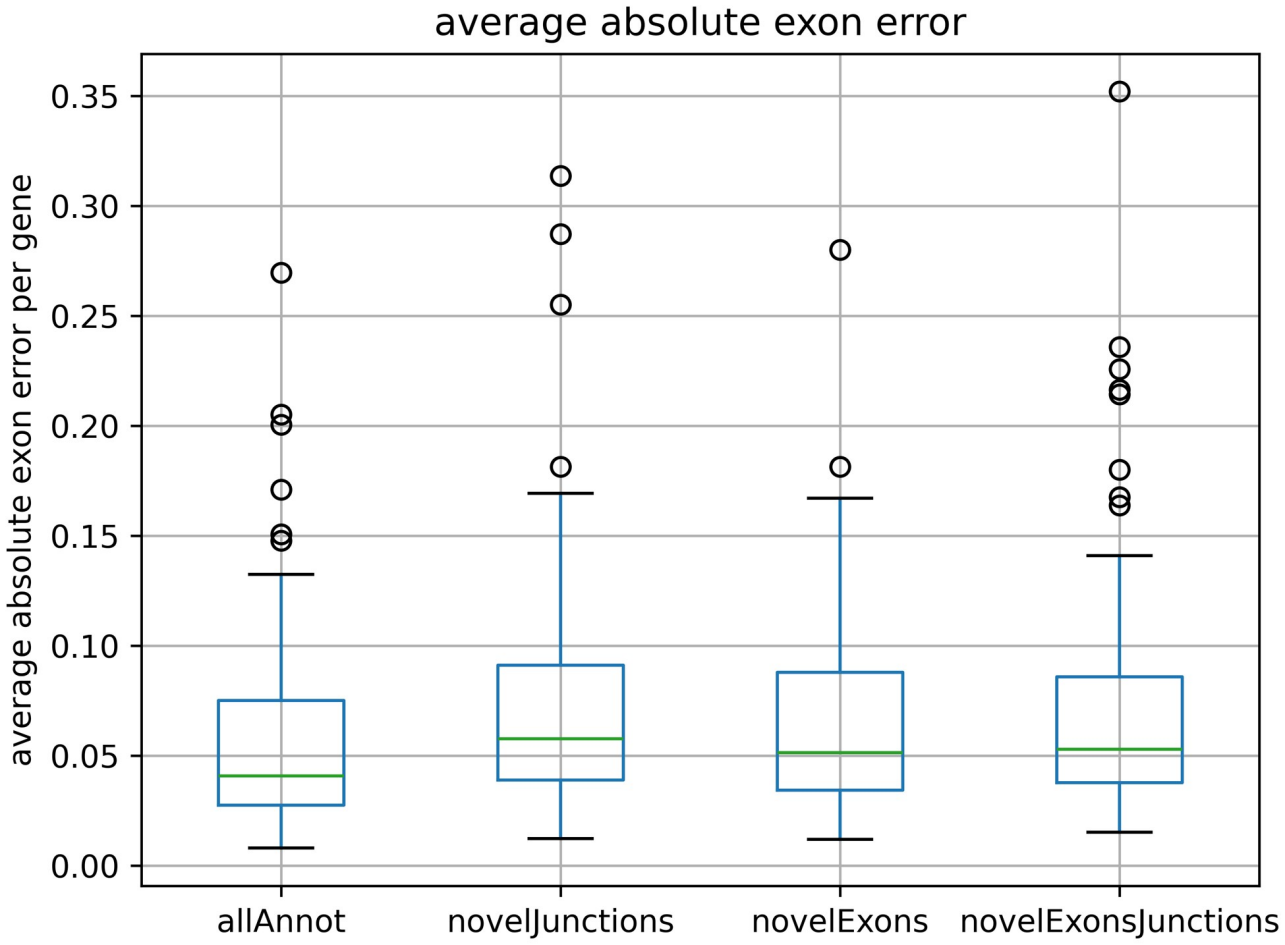
The distribution of the average absolute error for all genes. Box plots are made for each simulated dataset separately.

Figure 4 depicts the average absolute exon error per gene for each simulated dataset. In general, the estimated Ψ-values are accurate, even for datasets with novel splicing events, with most genes showing less than 5% absolute difference with their true Ψ-values. Additional accuracy metrics can be found in Supplementary Table S1. In Supplementary Material S4, we provide a thorough examination of factors that result in erroneous estimates of Ψ-values. The largest errors originate from small partial exons, which are exons that do not occur in any transcript as an exon by itself, but always occur together with their consecutive up- or downstream exon. Mapping reads to these partial exons is hard due to their short size, which leads to significantly underestimating their Ψ-values. Therefore, we advise users to be cautious when interpreting results for very short (partial) exons.

Besides the accuracy of the Ψ-values, the most important aspect of splicing analysis is the identification of differential splicing between (sub)types of cells. We simulated data for two groups of cells, each representing a different cell type with a distinct splice pattern. To detect differentially spliced exons between these simulated cell types, we calculated the difference Δ between the average Ψ-values of the two cell types. Genes with fewer than five cells per cell type remaining after ELLIPSIS filtering, are excluded, as there are not enough cells to reliably determine the average Ψ-values.

Exons are considered differentially spliced if they show a significantly high value of |Δ|. To establish a background distribution for Δ, we randomly reassign the cells to two cell types. As these cell types no longer represent groups of cells with distinct splice patterns, we do not expect differential splicing between them. We perform 10 random assignments, and fit a normal distribution to the resulting Δ-values. An exon is classified as differentially spliced if it is not likely to belong to this background distribution (*P*-value < .01, with Benjamini–Hochberg correction for multiple hypothesis testing).

For each simulated dataset, we compare the estimated $\Delta_{\text{est}}$ with the ground truth $\Delta_{\text{true}}$, and assess whether exons are correctly classified as differentially spliced. Table 1 shows that ELLIPSIS is able to accurately identify differentially spliced exons even when novel splicing events are present, with a precision between 0.79 and 0.94 and a recall between 0.54 and 0.74. More details can be found in Supplementary Tables S2–S5. The low recall mainly stems from exons that are only slightly differentially spliced ($|\Delta_{\text{true}}|<0.05$). These small differences are hard to distinguish from small errors in the estimated Ψ-values. Additionally, ELLIPSIS fails to detect most differentially spliced short (partial) exons, due to the underestimation of Ψ-values across all cells, making it difficult to identify potential differential splicing between cell types. This mapping bias arises from the limited exon length and affects all cells similarly.

**Table 1.** Accuracy metrics for the detection of alternatively spliced exons for ELLIPSIS and satuRn in each simulated dataset.

		ELLIPSIS	satuRn
Precision	allAnnot	0.79	0.78
	novelJ	0.93	0.78
	novelE	0.87	0.68
	novelEJ	0.83	0.71
Recall	allAnnot	0.66	0.58
	novelJ	0.50	0.45
	novelE	0.63	0.49
	novelEJ	0.51	0.43

All simulated datasets contain 100 genes, each with three annotated transcripts. The novelJ and novelE datasets contain an additional transcript with a novel junction resp. exon per gene. The novelEJ dataset contains two extra transcripts per gene, one with a novel junction and one with a novel exon.

#### 3.1.2 Identification of novel splicing events

We provide a more detailed analysis of the accurate identification of novel exons and junctions, as well as the reliability of detecting their differential splicing. Out of the 100 novel junctions introduced prior to read simulation in the novelJ dataset, 84 are identified and included in the extended splice graphs. Similarly, for the novelE dataset, 66 out of 100 novel exons are detected. For the novelEJ dataset, 86 out of 100 novel junctions and 70 out of 100 novel exons are identified by ELLIPSIS. As expected, novel exons are more challenging to identify, as both the incoming and outgoing junction connecting the novel exon to the rest of the graph need sufficient read coverage to enable adding the exon to the graph.

Despite this, ELLIPSIS still identifies more than half of the differentially spliced novel exons and junctions in the simulated datasets (recall ≥ 0.50) and only rarely misreports non-differentially spliced novel exons as differentially spliced (precision ≥ 0.94), see Supplementary Tables S6–S10.

#### 3.1.3 Comparison with state-of-the-art

We assess the performance of ELLIPSIS in comparison with Psix (Buen Abad Najar *et al.* 2022), BRIE2 (Huang and Sanguinetti 2021), and satuRn (Gilis *et al.* 2021), all methods designed for the detection of splicing in single cell data. None of them allow for the detection nor quantification of novel splice events: Psix only considers cassette exons from the reference genome, BRIE2 uses a list of predefined cassette exons, and satuRn relies on transcriptome mapping tools such as salmon-quant (Patro *et al.* 2017), which requires a list of known full length transcripts.

Psix is a probabilistic method that identifies cell state associated splicing of cassette exons in single cell data. It solely uses the coverage of splice junctions to find Ψ-values, and computes a Psix score using probabilistic modeling that represents the likelihood of the exon undergoing cell state-specific splicing. Psix, alike ELLIPSIS, relies on expression based intra-cell type similarity. However, unlike ELLIPSIS, Psix does not include any concept similar to conservation of flow, and is therefore bound to analyse each exon separately. Because Psix is restricted to the analysis of already annotated cassette exons, differential splicing in novel or more complex splicing events remains undetected.

BRIE2, uses a Bayesian regression model to identify cell type specific splicing. It uses the coverage of all exons and splice junctions involved in a splicing event, but as only cassette exons are considered, it only has a very local notion of conservation of flow that does not extend to neighboring splice events in the splice graph. Additionally, BRIE2 does not include intra-cell type similarity to increase its robustness.

Because both Psix and BRIE2 only analyse annotated cassette exons, we cannot directly compare their results with those of ELLIPSIS, which predicts results for all exons. To enable a fair comparison, Table 2 presents accuracy metrics calculated based solely on the annotated cassette exons considered by either Psix or BRIE2, with the specific cassette exons differing slightly between the two methods. Supplementary Tables S11 and S12 provide the absolute numbers of cassette exons used to derive these performance metrics. For the identification of differentially spliced cassette exons, ELLIPSIS shows equal or higher precision than Psix and BRIE2 for all datasets, at the expense of a seemingly lower recall. However, the lower recall of ELLIPSIS arises from evaluating performance on cassette exons alone, even though ELLIPSIS makes predictions for all exons. The latter includes exons that are more difficult to quantify, e.g. alternative start/end exons, and therefore necessitates a more stringent filtering strategy to guarantee reliable Ψ-estimates for all exons. ELLIPSIS therefore filters out genes for which most cells do not have sufficient coverage, and hence misses some differentially spliced cassette exons in those genes. Additionally, because ELLIPSIS examines a much larger set of exons (around 2000, compared to 80–100 for Psix and BRIE2) for differential splicing, it applies a more rigorous correction for multiple hypothesis testing. Both issues contribute to the observed lower recall for ELLIPSIS on cassette exons. However, given that cassette exons only constitute a minor fraction of the total number of exons that can be analysed, the recall for the identification of all types of differentially spliced exons of ELLIPSIS (≥0.50, see Table 1) is much higher than that of Psix and BRIE2 (around 0.02).

**Table 2.** Accuracy metrics for the detection of alternatively spliced casette exons for ELLIPSIS, Psix, and BRIE2 in each simulated dataset.

		ELLIPSIS	Psix	ELLIPSIS	BRIE2
Precision	allAnnot	0.90	0.85	0.91	0.85
	novelJ	1.00	0.86	0.93	0.87
	novelE	0.93	0.93	1.00	0.88
	novelEJ	0.95	0.89	0.94	0.85
Recall	allAnnot	0.69	0.85	0.54	0.87
	novelJ	0.44	0.77	0.36	0.85
	novelE	0.69	0.95	0.59	0.95
	novelEJ	0.50	0.80	0.40	0.79

Psix and BRIE2 each only consider certain cassette exons, therefore we only considered the same cassette exons for their comparison with ELLIPSIS. The allAnnot dataset contains 100 genes each with three annotated transcripts. The novelJ and novelE datasets both contain one additional transcript per gene, featuring either a novel junction (novelJ) or a novel exon (novelE). The novelEJ dataset contains two extra transcripts per gene, one with a novel exon and one with a novel junction.

Finally, we compare ELLIPSIS with satuRn. As satuRn performs differential splicing analysis at the isoform level, it inherently has some notions of conservation of flow, because exons are analysed jointly at the isoform level, rather than one by one. However, satuRn does not include information on intra-cell type similarity. In addition, because satuRn relies on transcriptome mapping to a known reference transcriptome, it does not allow detecting nor quantifying novel splicing events.

To compare the results of satuRn with ELLIPSIS, the predicted isoform-level Ψ-values were converted to exon-level Ψ-values. Table 1 shows that ELLIPSIS outperforms satuRn for the detection of alternatively spliced exons, with higher precision and recall for all simulated datasets. Interestingly, despite being presented as a tool for isoform-level splicing analysis, satuRn performs better when assessing the accuracy at exon level than at isoform level, see Supplementary Tables S13 and S14, emphasizing the challenges of aligning reads unambiguously to distinct transcripts.

### 3.2 Genome wide splicing analysis in glioblastoma

To show the applicability of our method, we analysed data from Darmanis *et al.* (GSE84465) (Darmanis *et al.* 2017). The dataset provides Smart-seq data on cells extracted from the tumor core and peripheral tissues of four glioblastoma patients, and therefore allows investigating the role of splicing during peripheral invasion in glioblastoma.

Using Seurat (Hao *et al.* 2024), we identified nine clusters of cells (see Supplementary Fig. S9), largely overlapping with the cell types identified by the authors: immune cells, oligodendrocyte progenitor cells (OPCs), oligodendrocytes, astrocytes, vascular cells, neurons, and neoplastic cells. All cell types are present in both the tumor core and in the periphery, except for astrocytes and neurons which are only found in the periphery. We focused on neoplastic and immune cells for further splicing analysis.

#### 3.2.1 Neoplastic cells

Most neoplastic cells are found in the tumor core, but some infiltrate the peripheral tissue, contributing to the aggressive nature and post-surgery relapse of glioblastoma (Mair *et al.* 2018, Li *et al.* 2020, Seker-Polat *et al.* 2022). To identify splicing differences between tumor core and peripheral cancer cells, we ran ELLIPSIS on the neoplastic cells (Supplementary Material S6.1). For each cell, we used the 100 most similar cells in terms of gene expression for the application of intra-cell type similarity. Because of their low number (63 out of 982 cells), the 100 most similar cells of neoplastic peripheral cells often originate from the tumor core. To prevent excessive smoothing of the Ψ-values for these rare peripheral neoplastic cells, we only considered the neighbors that originate from the same tissue (periphery versus tumor core). As such, we identified 993 genes that are differentially spliced between peripheral and tumor core neoplastic cells (Supplementary Table A).

In the presence of gene fusions, the conservation of flow property no longer holds, as the assumption that 100% of the transcripts pass through the source and sink of the splice graph of a gene is no longer valid. In that case, the two distinct splice graphs of the fused genes should be combined, instead of being analysed separately, and failing to do so, could result in erroneous detection of differential splicing. To assess whether for some genes such correction should be applied, we performed fusion detection with scFusion (Jin *et al.* 2022). Only one gene fusion (PPA2-ENSG00000251243) is detected, and neither of those genes contains an exon that is identified as differentially spliced. Although we cannot exclude that scFusion might have missed rare fusions, or fusions with low expression (Jin *et al.* 2022), no additional correction for the presence of gene fusions is applied.

When comparing differentially spliced genes with those that are differentially expressed (Supplementary Table C), we only observe a minor overlap (13 genes). Also at pathway level, the overlap is low: of the 27 GO pathways (Supplementary Table B) that are enriched amongst the 993 differentially spliced genes, and the 39 GO pathways (Supplementary Table D) enriched amongst the 281 differentially expressed genes, none overlap.

Gene set enrichment on genes that are differentially spliced between cancer cells originating from respectively the tumor core and the periphery, reveals pathways involved in cell migration, cell motility and locomotion, which is in line with the characteristics of the invasive peripheral cancer cells that migrate from the tumor core into the surrounding tissue. In addition, the enriched pathways related to respectively GTPase, cell adhesion and cytoskeleton organization are known to play a role in cancer cell invasion in glioblastoma (Friedl and Mayor 2017, Al-Koussa *et al.* 2020). Also pathways involved in the organization of cell projections (cilia and axons), assembly, and morphogenesis are identified, which are critical components of cell motility (Ashburner *et al.* 2000, Aleksander *et al.* 2023). Interestingly, the identification of the neuron projection morphogenesis pathway is in line with the findings of Venkataramani *et al.* (2022), who showed that invasive glioblastoma cells use mechanisms reminiscent of migration of immature neurons during brain development. These results show that splicing is an important regulator of the migratory behavior of glioblastoma cells, and acts independently of quantitative changes in gene expression.

To assess whether differentially spliced genes are co-regulated by common splice factors, we examined whether these differentially spliced genes were enriched in targets of respectively known splicing factors, using SpliceAid (Giulietti *et al.* 2013), and of RNA-binding proteins (RBPs) involved in splicing or the spliceosome, using clipDB (Zhao *et al.* 2022). For none of the splice factors from SpliceAid, targets were found significantly enriched amongst the differentially spliced genes. However, the same overrepresentation analysis, identified 40 RBPs with overrepresented targets amongst the differentially spliced genes (p.adjust < 0.05). From these RBPs, only HNRNPH1 is differentially expressed between tumor core and peripheral neoplastic cells, while four RBPs (HNRNPH1, HNRNPF, TRA2A, DDX42) are reported to be differentially spliced themselves. The latter suggests that alternative splicing of RBPs might, in turn, be responsible for alternative splicing of the genes they are interacting with.

#### 3.2.2 Immune cells

**Figure 5.**
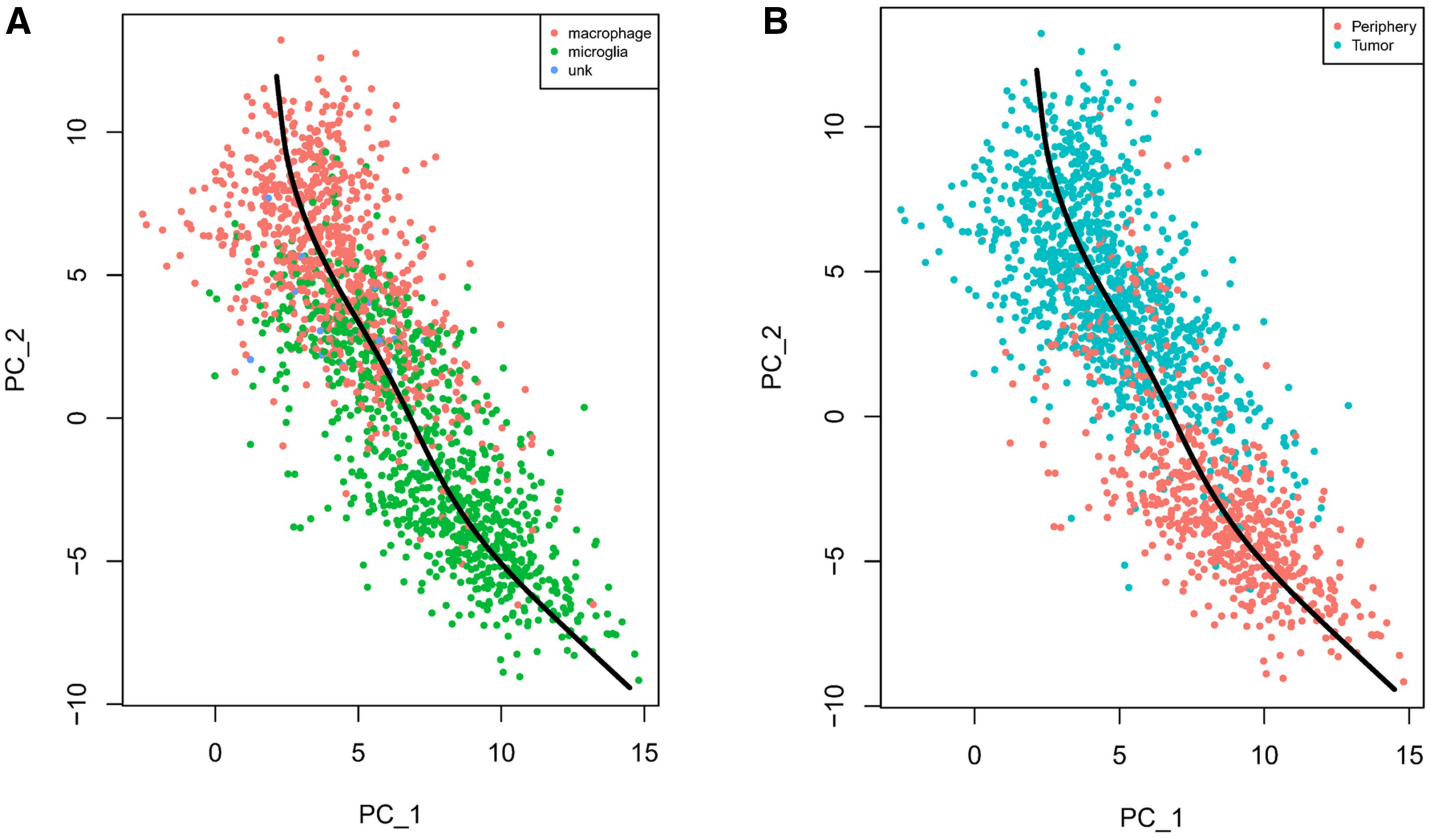
PCA plot for immune cells in Darmanis *et al.*, colored by (A) their type of immune cell, and (B) their tissue of origin.

The glioblastoma data also include immune cells from both the tumor core and peripheral tissue, see Supplementary Fig. S9. A trajectory analysis (Fig. 5B), reveals a gradual change between peripheral and tumor core immune cells. Interestingly, some peripheral immune cells show a “tumor core” like expression pattern, suggesting they could either be immune cells that originated in the tumor core and subsequently infiltrated together with the cancer cells to the tumor periphery. Alternatively, they could be immune cells residing in the periphery that underwent phenotypic changes after interaction with the infiltrating cancer cells.

In healthy brain tissue, immune cells are primarily microglia: tissue-resident macrophages of the central nervous system. However, under pathological conditions, bone-marrow derived macrophages might infiltrate the brain (Andersen *et al.* 2022). We discerned microglia-like from macrophage-like cells using the same approach as (Darmanis *et al.* 2017), and observed that the peripheral cells are enriched in microglia-like cells, while the tumor core cells contain more macrophage-like cells. However, as seen in Fig. 5A, there seems to be a gradual change from microglia-like to macrophage-like cells, rather than two distinct cell types.

To illustrate how ELLIPSIS can be used to identify differential splicing along a continuous process, we performed differential splicing analysis along the observed trajectory using ELLIPSIS (Supplementary Material S6.2). We identified 257 genes for which the Ψ-values are highly (anti-)correlated with pseudotime (Supplementary Table G). No GO biological processes were found enriched amongst these differentially spliced genes. In contrast, when performing a similar analysis for differentially expressed genes (Supplementary Tables E and F), several pathways related to response to stimuli, taxis, and cell migration were identified, indicating tumor-associated plasticity of immune cells driven by cell–cell communication with cancer cells. This again indicates that alternative splicing is a mechanism that acts independently of differential gene expression and that is much less characterized.

**Figure 6.**
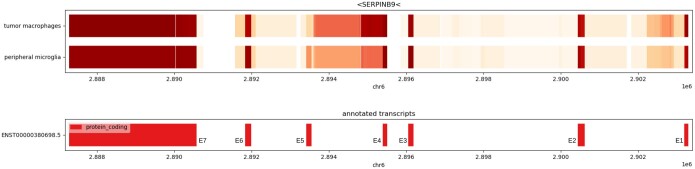
Visualization of average exon Ψ-values of gene SERPINB9 for the 10 most macrophage-like immune cells from the tumor core, and the 10 most microglia-like immune cells from the peripheral tissue. The macrophage-like cells show more intron retention, and less inclusion of exon 5.

As an illustration of a gene for which ELLIPSIS finds differential splicing along a continuous lineage of cells, we look at SERPINB9, a gene for which only one known transcript is annotated in the reference genome. ELLIPSIS reports a novel intron retention event between E4 and E5, that is more pronounced in macrophage-like cells, see Fig. 6. Additionally, in the macrophage-like cells the fifth exon is skipped more often than in the microglia-like cells. To ensure that the identified differences are biologically relevant and not due to coverage differences of SERPINB9-expression we analysed read coverage at the level of raw gene expression: a too low coverage in one of either cell type could obviate the detection of rare transcripts and result in the artefactual detection of differential splicing. Both analysed cell types show comparable coverages of SERPINB9 (Supplementary Fig. S2), with an average coverage that was only 1.13 times higher in the macrophages than in the microglia. This makes it unlikely that the detected splice differences are the result of coverage bias.

Remarkably, both splicing events observed in SERPINB9, i.e. E5-skipping and intron retention, introduce a premature stop codon. Premature stop codons could lead to nonsense mediated decay or truncated proteins (Lejeune 2022), hereby reducing the functionality of SERPINB9. As SERPINB9 protects cells from granzyme B induced apoptosis (Huang *et al.* 2024), its reduced functionality in macrophage-like cells, which are primarily found in the tumor core, can lead to increased apoptosis and hence a reduced immune response. Although further validation is needed to support this hypothesis, these results show how ELLIPSIS can identify novel splice variants with potential important implications in cancer.

## 4 Discussion

We introduced ELLIPSIS, a graph-based method designed for robust splicing quantification from Smart-seq data. ELLIPSIS capitalizes on intra-cell type similarity and conservation of flow principles to enhance Ψ-value accuracy. A unique asset of ELLIPSIS is its capacity to identify and quantify previously unannotated splice variants with high reliability.

Using simulated data, we showed how ELLIPSIS identifies most novel exons and junctions and adds them to the splice graphs, allowing for the accurate estimation of Ψ-values for genes with and without novel splicing events. Because ELLIPSIS estimates Ψ-values for all exons and junctions in the splice graphs, is not limited to the five common types of splice variants in Supplementary Fig. S1, but allows for the quantification of more complex splice variants. For example, in Fig. 2B, exons E1–E4 can form three different splice variants: E1–E4, E1–E3–E4, and E1–E2–E3–E4; which does not fit within a single common splice category. In addition, by including artificial source and sink exons in the splice graphs, ELLIPSIS allows for the quantification of alternative first/last exon usage, which is an often overlooked type of alternative splicing that can affect translational efficiency, mRNA stability and transcript function (Elkon *et al.* 2013, Ushijima *et al.* 2017).

Using simulated data, we showed that ELLIPSIS outperforms Psix (Buen Abad Najar *et al.* 2022), BRIE2 (Huang and Sanguinetti 2021), and satuRn (Gilis *et al.* 2021) for the identification of differential splicing, especially for novel or more complex splice variants.

To illustrate on a real dataset how ELLIPSIS can detect differential splicing between distinct groups of cells or along a trajectory, we applied it to the glioblastoma single cell dataset of Darmanis *et al.* (2017). Gene set enrichment on genes that are differentially spliced between tumor cells from the core versus. the periphery, identified pathways involved in cell migration and motility, which is in line with the characteristics of invasive peripheral cancer cells that migrate from the tumor core into the surrounding tissue. Comparing the genes that are differentially spliced with those that are differentially expressed, resulted in a minor overlap both at gene and pathway level. This corroborates previous findings that showed that differential splicing mediates distinct biological processes compared to differential gene expression (Stilling *et al.* 2014, Dominguez *et al.* 2016, Li *et al.* 2016, Girardot *et al.* 2018, Gilis *et al.* 2021, Glinos *et al.* 2022, Dam *et al.* 2023, García-Pérez *et al.* 2023). In addition, we found differentially spliced genes involved in neuron projection morphogenesis, indicating that splicing might play a role in hijacking this developmental mechanism for cancer cell infiltration.

Correlating differential splicing with trajectory analysis of the immune cells in glioblastoma showed that many genes that are potentially involved in driving gradual changes in cell characteristics are not easily described at pathway level.

## 5 Conclusions

We present ELLIPSIS, a tool to quantify alternative splicing from Smart-seq based single-cell RNA-seq data. ELLIPSIS is unique in handling complex splice variants and discovering and quantifying novel, not yet annotated, splicing events at the single cell level. It enables the robust quantification of Ψ-values by combining local read coverage with conservation of flow and intra-cell type similarity, managing the low and uneven coverage inherently present in short read scRNA-seq data.

## Supplementary Material

btaf028_Supplementary_Data
