# Supplementary material for: ELLIPSIS: robust quantification of splicing in scRNA-seq
Source: Bioinformatics. 2025 Feb 12;41(2):btaf028. doi: 10.1093/bioinformatics/btaf028 (PMC11878791; doi:10.1093/bioinformatics/btaf028)
Supplement: btaf028_Supplementary_Data [file btaf028_supplementary_data.zip › aa61b_paperSplicingSupplementary.pdf]

Supplementary Material

1 Supplementary Figures

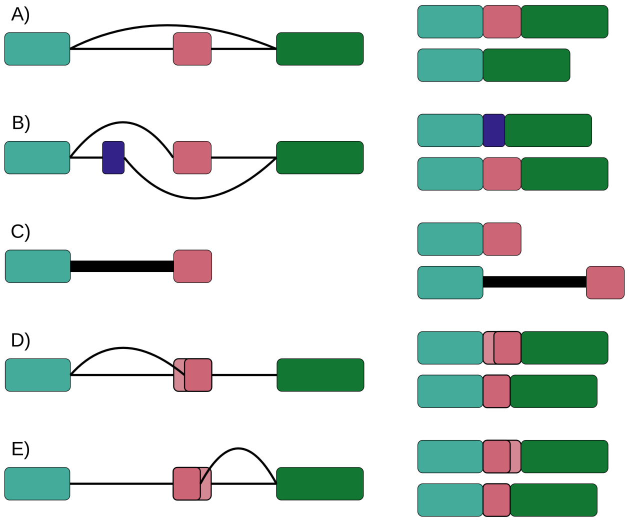

Fig. S1: The 5 common types of splice variants. A) cassette exon B) mutually exclusive exons C) retained exon D) alternative 5' end splicing E) alternative 3' end splicing

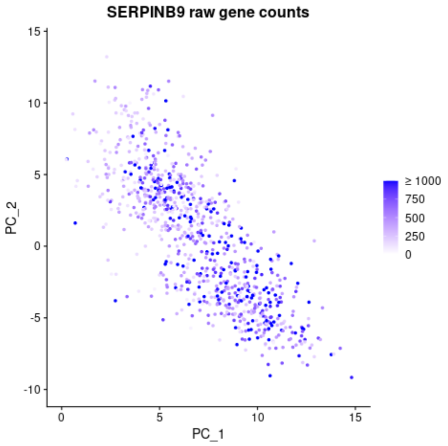

Fig. S2: Raw gene counts of SERPINB9 in the immune cells.

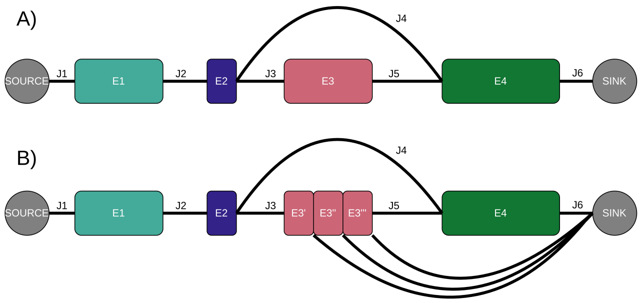

Fig. S3: Two similar splice graphs. In graph A), exon E3 is one large exon, while in graph B) exon E3 is subdivided into 3 distinct smaller exons (E3', E3" and E3"') due to alternative 3' splicing.

2 Supplementary Tables

	allAnnot	novelJ	novelE	novelEJ
number of genes computed	95	96	95	93
min exonError	0.007889	0.012192	0.011809	0.015116
median exonError	0.040733	0.057685	0.051348	0.052806
mean exonError	0.059229	0.073304	0.064304	0.072428
max exonError	0.269498	0.313657	0.279957	0.351934

Table S1: Exon accuracy metrics for different types of simulated data, each containing reads for 100 genes. The allAnnot dataset contains 3 annotated transcripts per gene, the novelJ and novelE datasets both contain one additional transcript per gene, featuring either a novel junction (novelJ) or a novel exon (novelE). The novelEJ dataset contains two extra transcripts per gene, one with a novel exon, and one with a novel junction. The number of genes computed are the total number of genes that passed the coverage filters of ELLIPSIS. The exonError is defined per gene as the average absolute difference between the estimated and true Ψ-values across all base pairs contained in the exons of the corresponding splice graph. For each dataset, we show the minimum, median, mean and maximum exonError per gene.

**allAnnot**		estimated	
		diff. spliced	not diff. spliced
true	diff. spliced	296 (TP)	152 (FN)
	not diff. spliced	80 (FP)	1695 (TN)

Table S2: Confusion table comparing true and estimated differential splicing of all exons using the simulated dataset with only annotated transcripts. 45% of false negatives are only slightly differentially spliced, with $\Delta_{\text{true}} \in [-0.08, 0.08]$. Precision = 0.79, Recall = 0.66

**novelJ**		estimated	
		diff. spliced	not diff. spliced
true	diff. spliced	275 (TP)	271 (FN)
	not diff. spliced	21 (FP)	1292 (TN)

Table S3: Confusion table comparing true and estimated differential splicing of all exons using the simulated dataset with novel junctions. 67% of false negatives are only slightly differentially spliced, with $\Delta_{\text{true}} \in [-0.10, 0.10]$. Precision = 0.93, Recall = 0.50

novelE		estimated	
		diff. spliced	not diff. spliced
true	diff. spliced	331 (TP)	213 (FN)
	not diff. spliced	51 (FP)	1650 (TN)

Table S4: Confusion table comparing true and estimated differential splicing of all exons using the simulated dataset with novel exons. 44% of false negatives are only slightly differentially spliced, with $\Delta_{\text{true}} \in [-0.08, 0.08]$. Precision = 0.88, Recall = 0.63

novelEJ		estimated	
		diff. spliced	not diff. spliced
true	diff. spliced	334 (TP)	318 (FN)
	not diff. spliced	69 (FP)	1716 (TN)

Table S5: Confusion table comparing true and estimated differential splicing of all exons using the simulated dataset with novel exons and junctions. 55% of false negatives are only slightly differentially spliced, with $\Delta_{\text{true}} \in [-0.07, 0.07]$ Precision = 0.83, Recall = 0.51

dataset	novel	precision	recall
novelJ	junctions	1	0.56
novelE	exons	0.94	0.50
novelEJ	junctions	1	0.58
novelEJ	exons	1	0.59

Table S6: Precision and recall for differential splicing detection of novel exons/junctions in the simulated datasets. More than half of the differentially spliced novel exons and junctions are correctly detected. Only the novelE dataset has 1 novel exon that is misreported as differentially spliced.

novelJ		estimated		
		diff. spliced	not diff. spliced	not included
true	diff. spliced	18 (TP)	14 (FN)	2
	not diff. spliced	0 (FP)	34 (TN)	6

Table S7: differential splicing identification of novel junctions in the novelJ simulated dataset. Precision = 1, Recall = 0.56

	novelE	estimated		
		diff. spliced	not diff. spliced	not included
true	diff. spliced	17 (TP)	20 (FN)	8
	not diff. spliced	1 (FP)	35 (TN)	11

Table S8: differential splicing identification of novel exons in the novelE simulated dataset. Precision = 0.94, Recall = 0.50

	**novelEJ**	estimated		
	(junctions)	diff. spliced	not diff. spliced	not included
true	diff. spliced	20 (TP)	14 (FN)	3
	not diff. spliced	0 (FP)	41 (TN)	6

Table S9: differential splicing identification of novel junctions in the novelEJ simulated dataset. Precision = 1, Recall = 0.59

	**novelEJ**	estimated		
	(exons)	diff. spliced	not diff. spliced	not included
true	diff. spliced	18 (TP)	13 (FN)	5
	not diff. spliced	0 (FP)	53 (TN)	13

Table S10: differential splicing identification of novel exons in the novelEJ simulated dataset. Precision = 1, Recall = 0.58

3 Smart-seq2 read simulation

To assess the accuracy of the reported Ψ-values by ELLIPSIS, we simulate Smart-seq2 data for several genes with known splice patterns. We use the Polyester package to simulate raw reads of genes with and without differential splicing. Reads are simulated for 2 clusters of 100 cells each, with the 2 clusters representing distinct cell types. For all cells, reads are generated for 50 genes that are differentially spliced between the 2 clusters, and 50 genes that are non-differentially spliced as negative controls.

First, we randomly select 100 genes, and for each gene we choose 3 already annotated transcripts. An example of such gene can be found in Fig. 3A. Optionally, additional transcripts are created by introducing novel splice junctions and/or exons. Novel splice junctions are added between 2 already annotated exons, which results in a novel transcript (see Fig. 3B). However, when a novel exon is introduced, there are also 2 additional junctions that need to be introduced to connect the novel exon to the rest of the splice graph, as can be seen in Fig. 3C. We create the novel exon such that these novel junctions are canonical. Thus, the novel exon is flanked with AG at its 5' end, and GT at its 3' end. We also generate a dataset where we introduce one novel junction and one novel exon in each gene, which results in a combination of Fig. 3 B and C.

Differentially spliced genes are simulated by considering different mixtures of the aforementioned transcripts, where each cluster has distinct proportions of transcripts. In the negative control genes, we still simulate reads for all selected transcripts of that gene, but we assume that they are present in the same proportions in both clusters, as such, they are not differentially spliced.

To simulate reads for mixtures of transcripts with different proportions, we compute the number of reads for transcript t from gene g in cell c of cluster d, as follows:

$$\text{numReads}(t, c) = \text{cellSize}(c) \cdot \text{geneExpression}(g, d) \cdot \text{freq}(f, d) \tag{1}$$

Where cellSize is a cell-specific random value representing the total amount of RNA in cell c. In single cell data, some cells are more deeply sequenced than others, which is represented by the cellSize parameter. The geneExpression represents the total gene expression for gene g in cluster d. Because we assume that gene expression is cluster-specific, the gene expression values are fixed throughout the different cells contained in the same cluster. Finally, freq represents the frequency of transcript t in all transcripts of its corresponding gene. Based on the intra-cell type similarity assumption, splicing is cluster specific. Therefore the value of freq is fixed throughout the different cells contained in the same cluster. For the genes that are not differentially spliced (negative controls), the frequency values are the same for all cells in both clusters.

Next, we use the resulting number of reads to simulate an experiment with Polyester using parameters to maximally emulate Smart-seq2 data. To assess

the technical biases present in such data, we use the dataset from Darmanis et al. [3], in which we randomly subsampled 1000 cells to speed up computation time. This dataset includes 92 ERCC transcripts, which can be used to examine protocol biases, as they are not impacted by splicing or other biological processes.

First, we analyzed the GC bias present in the reads mapping to any ERCC transcript. Because we did not observe a GC-bias in the ERCC transcripts, we did not add any GC-bias to the Polyester-simulated data. Even for ERCC-transcripts with average GC-content relatively far from 50%, the distribution GC-content of the reads mapping to that transcript remains balanced, see Fig. S4 for an example of such ERCC transcript.

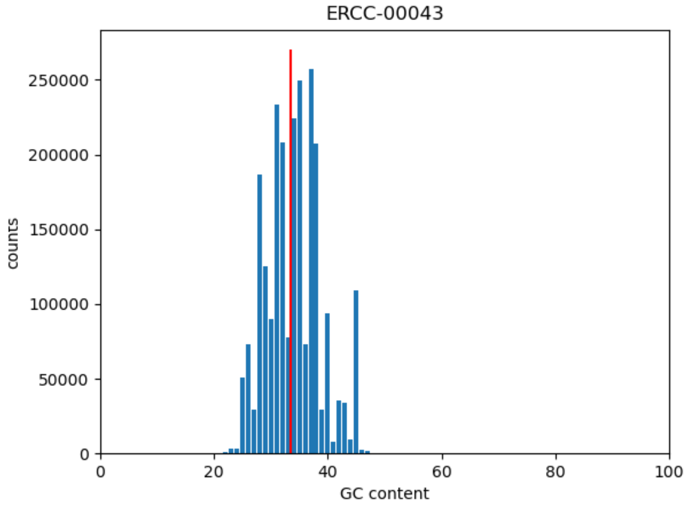

Fig. S4: The distribution of GC-content of all reads mapping to transcript ERCC-00043. The GC-content of the entire transcript itself is indicated by the red line.

Additionally, Archer et al. [1] describe the coverage bias in transcripts as a result of non ideal poly-A tagging and non ideal template switching during the Smart-seq2 library preparation. This coverage bias depends on the total length of the transcript, and mainly affects long transcripts (> 4kb). As the ERCC transcripts are relatively short (0.5 - 2 kb), we did not see such positional bias in the ERCC transcripts. However, because most genes have longer transcripts that will suffer from this positional bias, we use the positional bias as described by model B&D in [1], which corresponds to Smart-seq2 library preparation.

Next to the positional bias due to non ideal poly-A tagging and template switching, we also see a decrease in coverage at the end of the ERCC transcripts due to Illumina purification, which removes extremely short fragments resulting from tagmentation near the ends of the RNA transcripts. We did not include

this end-of-transcript bias when simulating reads with Polyester, as Polyester automatically filters short fragments and thus inherently adds this negative selection bias.

Additionally, we obtain the protocol-specific parameters, fragment distribution (see Supplementary Fig. S5) and read length (75 bp), based on the the observations for the ERCC transcripts.

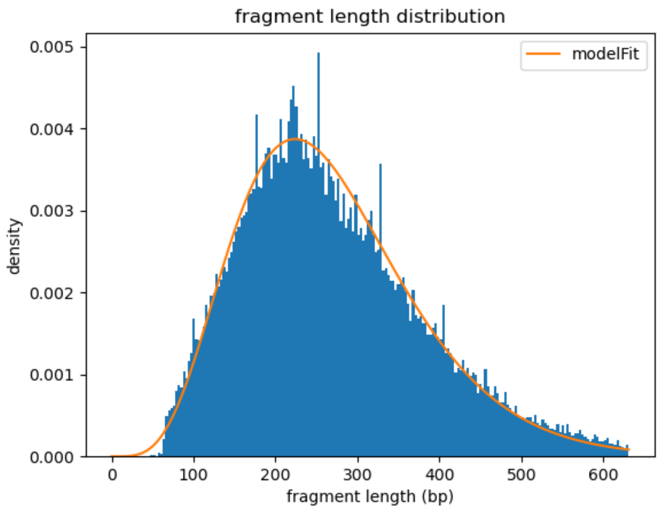

Fig. S5: The distribution of fragment lengths, or number of basepairs between both ends of a paired-end read (incl. reads itself), for the reads mapping to the ERCC transcripts in Darmanis et al. [3].

Finally, the the resulting reads go through the same preprocessing pipeline as real data: STAR alignment, gene level counting with HTSeq-count, and neighbor determination using Seurat [6]. Subsequently, we run ELLIPSIS, where we use the 20 closest neighbors for the intra-celltype similarity equations. We keep this number of neighbors rather small, to emulate the limited number of useful neighbors in real datasets, where quite some neighboring cells are filtered due to low gene expression or low quality mapping.

4 Distribution of errors made by ELLIPSIS

We use the simulated data to compare the obtained Ψ-values with the ground truth Ψ-values to assess for which types of exons ELLIPSIS makes the largest mistakes. Hereto, we assess whether erroneous Ψ-estimates made by ELLIPSIS are correlated to the ground truth Ψ-values. In Fig. S6, we see that the majority of errors, defined as $\Psi_{\text{true}} - \Psi_{\text{est}}$, particularly those from long exons, are

small. However, for many small exons, the error on Ψ_{est} is maximally negative, i.e. ELLIPSIS predicts $\Psi_{\text{est}} = 0$, while $\Psi_{\text{true}} > 0$. These small exons are typically partial exons: exons that do not occur in any transcript as an exon by themselves, but always occur together with their consecutive up- or downstream exon. These partial exons can be very short, only a few nucleotides long, which makes them harder to map to. As a result, no or only very few reads map to these small exons, resulting in low Ψ_{est}.

In Fig. S7, E2' is an example of a small partial exon that occurs as an extension of E2. In the corresponding transcripts, you would find only exon E2, or the concatenation of exons E2' and E2. Exons E1 and E2 are sufficiently long, and will therefore have read coverage proportional to their Ψ_{true}-values, while E2' will have low or even no coverage due to its short length, which also causes J1 and J3 to have low/no read coverage. As a result, ELLIPSIS will assign high Ψ_{est} for J2, instead of J1, E2' and J3.

For differential splicing detection, this does not result in low precision, as the effect is the same for all cells. However, it does partially explain the low recall, as ELLISPIS reports $\Psi_{\text{est}} = 0$ in many short partial exons for all cells, obviating the identification of differential splicing between cell types in these exons.

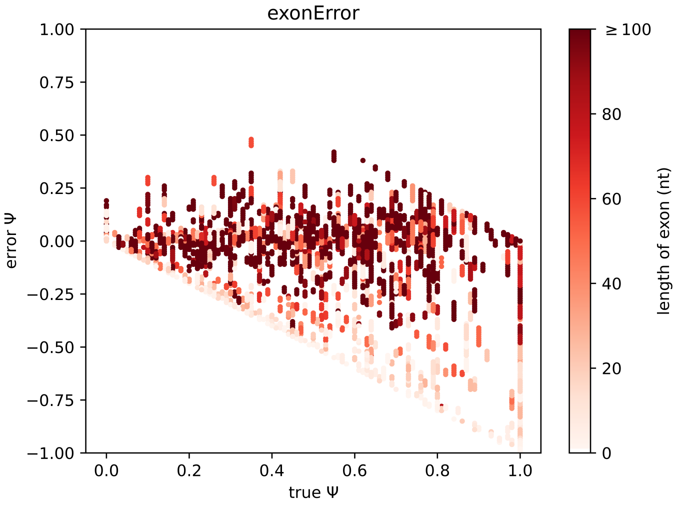

Fig. S6: The distribution of the exon error made by ELLIPSIS, defined as $\Psi_{\text{true}} - \Psi_{\text{est}}$, as a function of the true Ψ-values, for the allAnnot simulated dataset.

Next, we assessed whether the average error made by ELLIPSIS is correlated to the value of Ψ_{true}, and if capping the values for Ψ_{est} at 0% and 100% reduces the error around $\Psi = 0\%$ or $\Psi = 100\%$. Hereto, we gathered all exons with the same Ψ_{true}-value, and computed the weighted average of the absolute error

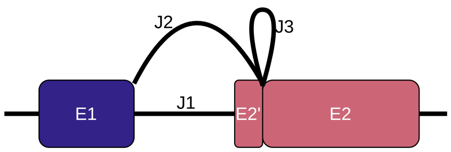

Fig. S7: A part of a splice graph with partial exon E2'. This exon is an extension of exon E2.

made by ELLIPSIS. We used the exon lengths as weights. In Fig. S8, we see that the average absolute error increases with increasing Ψ_{true}, which can be attributed to small partial exons that show larger absolute errors when Ψ_{true} is larger. Capping Ψ_{est} at 0% and 100%, does not significantly reduce the error around $\Psi = 0\%$ or $\Psi = 100\%$, indicating that the conservation of flow works. Due to the conservation of flow, exon errors are not independent from each other, meaning that capping the Ψ-value of a certain exon at 0% or 100%, also influences its neighboring exons. We use the example of an exon that would have $\Psi_{\mathrm{est}} > 100\%$ due to locally increased coverage, but due to capping, we estimate $\Psi_{\mathrm{est}} = 100\%$. By reducing this value, we also have to reduce the values for its neighboring exons in the graph to still comply with the conservation of flow, which actively reduces the error for those exons as well. As a result we do not observe artificially decreased errors near the extremities of Ψ_{true}

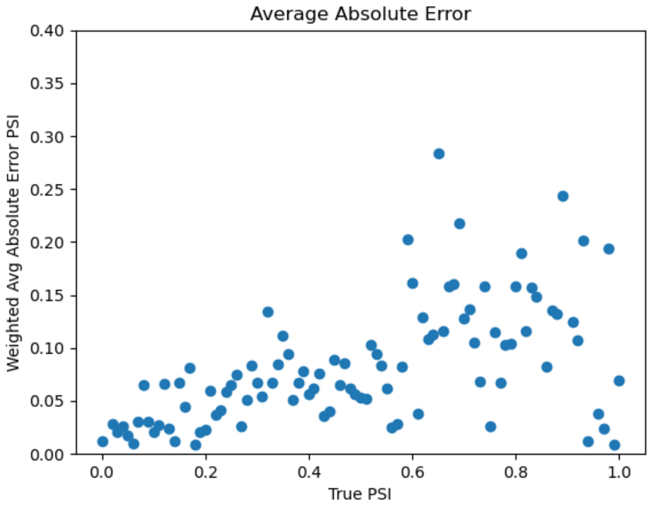

Fig. S8: the average absolute exon error made by ELLIPSIS in function of the true Ψ-values for the allAnnot simulated dataset

5 Comparison with state-of-the-art

5.1 Comparison with Psix

Next to comparing the obtained Ψ-values with the ground truth Ψ-values for the simulated data, we also compare the differentially spliced genes that were obtained using the results form ELLIPSIS, with a state-of-the art differential splicing tool, Psix [2]. Psix requires a latent space for cell similarity metrics. Hereto, we applied the standard Seurat pipeline [6]: normalization, scaling and PCA reduction, and used the latent spaced defined by the first 50 principle components. Because the simulated dataset is rather small (200 cells) we use *n_neighbors* = 20, which is the same as we used for ELLIPSIS. Cassette exons are considered differentially spliced when their p-value < 0.05.

In contrast to ELLIPSIS, Psix only examines cassette exons that occur in the reference annotation. The number of such cassette exons in our simulated dataset is small versus the total number of exons: 81 in allAnnot, 80 in novelJ, 78 in novelE, and 79 in novelEJ. To allow for a fair comparison, we therefore restrict the benchmarking to the detection of differential splicing in the cassette exons defined by Psix.

Table S11 the performance metrics for Psix and ELLIPSIS. ELLIPSIS shows slightly better precision in identifying the ground truth differentially spliced cassette exons, while Psix shows a seemingly better recall. This lower recall is explained by the more stringent gene filtering and multiple hypothesis correction applied by ELLIPSIS. To obtain reliable Ψ-estimates for all exons, ELLIPSIS filters out genes for which most cells have low read coverage. As a result, exons in those genes are labeled as false negatives, even though ELLIPSIS does not make any (wrong) prediction for these exons. Additionally, the results of ELLIPSIS are corrected for multiple hypothesis testing with around 2000 exons, while Psix only corrects for around 80 tests. Interestingly, both ELLIPSIS and Psix show low recall for the novelJ dataset, mainly due to the higher prevalence of differentially spliced cassette exons with small Δ_{true}, which are harder to detect by both methods.

Given that the cassette exons only comprise a minor fraction of the total number of exons in the dataset, the true recall of Psix is largely overestimated by only using the cassette exons as ground truth. In total, ELLIPSIS correctly identified around 300 differentially spliced exons in each simulated dataset, while Psix identified only around 35 differentially spliced cassette exons. When considering all exons that could be identified as differentially spliced in the simulated datasets, the recall of Psix could be around 0.02 in all simulated datasets, whereas ELLIPSIS has a recall ≥ 0.50 (see Table S2-5).

5.2 Comparison with BRIE2

We also compared the detection of differentially spliced exons with BRIE2 [7]. In theory, BRIE2 allows for the identification and quantification of al alternative splicing events that have 2 distinct splice options, which includes all common

		allAnnot	novelJ	novelE	novelEJ
cassette exons analyzed		81	80	78	79
Psix	TP	33	30	37	32
	FP	6	5	3	4
	TN	36	36	36	35
	FN	6	9	2	8
	precision	0.85	0.86	0.93	0.89
	recall	0.85	0.77	0.95	0.80
ELLIPSIS	TP	27	17	27	20
	FP	3	0	2	1
	TN	39	41	37	38
	FN	12	22	12	20
	precision	0.90	1.00	0.93	0.95
	recall	0.69	0.44	0.69	0.50

Table S11: metrics comparing Psix with ELLIPSIS for the cassette exons identified by Psix.

types of splice events (Fig S1). However, generating a splice event annotation file, requires BRIEkit (https://github.com/huangyh09/briekit/wiki), which we were unable to install due the use of currently unsupported or outdated versions of python (2.7) and libgfortran (3). Therefore, we used the most recent human splice event annotation available on sourceforge (gencode.v27), which only contains cassette exons.

For each simulated dataset, we run *brie-count* with default parameters, followed by *brie-quant* in mode2 to find differential splicing between the 2 cell types, with *–interceptMode gene*. Cassette exons are considered differentially spliced if their FDR < 0.05.

For this comparison, we only consider the cassette exons that were included in the splice event annotation from BRIE2 and are considered expressed by BRIE2. In Table S12, we see that ELLIPSIS has better precision, but seemingly lower recall than BRIE2 for these exons. Similarly to Psix, the lower recall is largely caused by the more stringent coverage filtering and the vast difference in number of exons tested (2000 vs 100 tests). If all differentially spliced exons would be considered, the recall of BRIE2 would be around 0.02, whereas the one of ELLIPSIS is ≥ 0.50.

5.3 Comparison with satuRn

We compare the results from ELLIPSIS with those of satuRn [4], which is a method to identify differential isoform usage between different groups of cells. In contrast to ELLIPSIS, it does not allow for the analysis of differential splicing along a continuous process, such as a trajectory. SatuRn performs an analysis at the full isoform level, instead of looking at the exon/junction level. Hereto, it requires a transcriptome alignment, which was performed with *salmon-quant*

		allAnnot	novelJ	novelE	novelEJ
cassette exons analyzed		97	99	101	104
BRIE2	TP	34	33	37	33
	FP	6	5	5	6
	TN	52	55	57	56
	FN	5	6	2	9
	precision	0.85	0.87	0.88	0.85
	recall	0.87	0.85	0.95	0.79
ELLIPSIS	TP	21	14	23	17
	FP	2	1	0	1
	TN	56	59	62	61
	FN	18	25	16	25
	precision	0.91	0.93	1.00	0.94
	recall	0.54	0.36	0.59	0.40

Table S12: metrics comparing BRIE2 with ELLIPSIS for the cassette exons identified by BRIE2.

[8] in alignment-based mode using default arguments.

After running satuRn on the transcriptome alignment, it appears to detect many isoforms not included in the simulated data, showing that transcriptome alignment of short reads is still not trivial. Many reads map to exons shared by different isoforms, resulting in ambiguous alignments.

First, we computed accuracy metrics for the identification of differential isoform usage. As proposed in by the authors, we use empirical_FDR < 0.05 to detect differential isoform usage. Table S13 shows that satuRn has a low precision for all simulated datasets (< 0.54), which is largely due to the spurious detection and quantification of non-expressed isoforms. The recall drops drastically for the simulated datasets that include novel exons and/or junctions, because the isoforms containing novel splicing events were not included in the reference genome, and therefore not in the satuRn analysis.

dataset	precision	recall
allAnnot	0.54	0.88
novelJ	0.57	0.53
novelE	0.53	0.59
novelEJ	0.45	0.40

Table S13: Precision and recall for isoform-level differential splicing detection in the simulated datasets for satuRn

To enable a comparison with ELLIPSIS, we converted the isoform level Ψ-values from satuRn to exon level Ψ-values. For each exon, the Ψ-value corresponds to the sum of predicted Ψ-values for all transcripts that include that

exon. Interestingly, although satuRn is an isoform level tool, the exon-level analysis provides a higher precision, see Table S14. We see again a decrease in recall for the datasets that include novel splicing events, although less pronounced than for the isoform-level analysis. When compared to the results in Tables S2-S5, we see that ELLIPSIS has a better performance than satuRn at the exon-level.

dataset	precision	recall
allAnnot	0.78	0.58
novelJ	0.78	0.45
novelE	0.68	0.49
novelEJ	0.71	0.43

Table S14: Precision and recall for exon-level differential splicing detection in the simulated datasets for satuRn

6 Analysis of glioblastoma data

Raw data is obtained from Darmanis et al. [3] (GSE84465). The dataset consists of 3589 cells from 4 patients with IDH1-negative, grade IV GBMs. For each patient, cells are obtained from the tumor core and the surrounding peripheral tissue. Both unpurified cells and populations enriched for the major CNS celltypes (neurons, astrocytes, myeloid cells, endothelia) were collected.

Reads are aligned to the reference genome (human Gencode GRCh28 primary assembly) using STAR in 2-pass mode. Because the data contains ERCC transcripts, the ERCC92 reference from thermofisher was added to the human reference genome. After alignment, all non-primary alignments, unmapped reads and reads without a proper pair are removed as they take up unnecessary computation time in later steps. Additionally, the reads mapping to the ERCC transcripts are removed prior to performing alternative splicing detection.

Next, gene level counts are computed using HTSeq-count using parameters *–stranded no* and *–mode intersection-nonempty*. Based on the gene expression values, Seurat [6] is used to determine cell-type clusters and identify the closest neighbors for each cell. To ensure a minimal data quality for further downstream analysis, 62 cells with less than 500 expressed genes or less than 10 000 reads are removed. Additionally, 154 cells with more than 25% mitochondrial reads are removed, as they are more likely to be dead cells.

For the remaining cells, we follow the standard Seurat pipeline of normalization, selecting highly variable features, scaling (while regressing out the percentage of mitochondrial reads) and PCA reduction. We select the 25 first PC's and perform findNeighbors and findClusters. This results in 9 clusters, which greatly overlap with the cell types described in the original paper (see Fig. S9. Based on the gene expression of the marker genes for each of the celltypes in the original paper, we label the clusters identified by Seurat as immune cells 1,

neoplastic cells 1, immune cells 2, OPCs, neoplastic cells 2, oligodendrocytes, astrocytes, vascular cells and neurons.

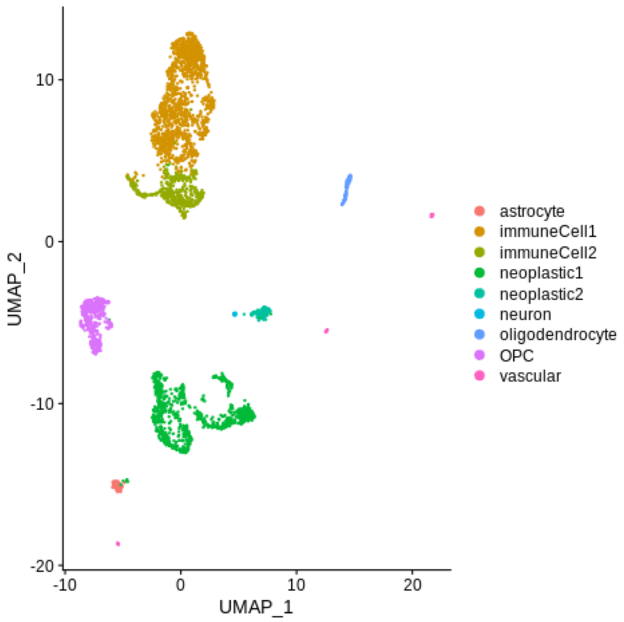

Fig. S9: UMAP representation of all cells in [3] after clustering by Seurat.

Next, we selected 2 different subsets of cells: the neoplastic cells (both neoplastic clusters) and the immune cells (both immune cell clusters). For each of these subsets, we perform differential expression with the *FindMarkers* function in Seurat, and differential splicing analysis with ELLIPSIS.

6.1 Neoplastic cells

We selected both neoplastic clusters, which results in 982 cells. Using only those cells, we selected the 2000 most variable genes and performed PCA dimensionality reduction. Next, the 100 closest neighbors of each cell are determined using FindNeighbors with the 20 first PCs. To obtain an optimal separation between tumor core and peripheral neoplastic cells, we filtered out the neighbors that did not originate from the same tissue (resp. tumor core and periphery).

We perform differential splicing analysis with ELLIPSIS, using default parameters except for $maxIter = 500$ and $maxPaths = 10^{15}$, to ensure most genes converge and more complex genes are included. When filtering with default $maxPaths = 10^6$, 8% of genes are filtered out, while only 0.04% of genes are removed when $maxPaths = 10^{15}$.

The resulting PSI values are used to identify differentially spliced exons for all genes for which Ψ-values are computed in at least 5 cells in both groups. The background distribution of the difference Δ between both imbalanced groups, is estimated by randomly assigning the cells to a tissue of origin, with the same size imbalance (63 peripheral and 919 tumor core cells). We generate 10 random reassignments, and compute the Ψ-values for 100 random genes for each assignment. Next, we compute the difference Δ in average Ψ-values between the 2 groups, and fit a normal distribution.

An exon is considered as differentially spliced if it is not likely to belong to this background distribution (p-value < 0.05, with Benjamini-Hochberg correction for multiple hypothesis testing). We perform Pathway analysis using ClusterProfiler [9], considering only genes for which Ψ-values are computed in at least 5 cells per group, as background.

Differential gene expression analysis between tumor core and periphery cells is performed using Seurat *FindMarkers*, and significant genes are selected with adjusted p-value < 0.05, and log2FC > 1. Pathway analysis on the resulting genes is performed using ClusterProfiler [9], using all genes as background.

Using spliceAid [5] and clipDB [10] databases, we analyzed if there are splice factors that simultaneously regulate splicing of multiple genes. Hereto, we used a hypergeometric test to compute the p-value for the over-representation of differentially spliced genes interacting with each splice factor, and used Benjamini-Hochberg correction to adjust the p-values for multiple hypothesis testing.

6.2 Immune cells

For the analysis of the immune cells, we used the subset of cells in both immune cell clusters (1785 cells in total). Using only those cells, we selected the 2000 most variable genes and reduced the dimensions using PCA on those 2000 genes. Using the top 2 PCs, the immune cells were reclustered into 4 clusters using *hclust (method = "ward.D2")*, and *cuttree*. Next, a trajectory between those clusters is fitted using slingshot, where we used the cluster containing most peripheral cells as origin as those can be considered normal cells.

Differential gene expression of the 2000 most variable genes along this trajectory is performed by tradeSeq, correcting for patient effects. We analyzed the correlation of gene expression with pseudotime using the *associationTest*-function from TradeSeq, and the difference in expression between start and end using *startVsEndTest*. Genes are considered differentially spliced along the trajectory if the adjusted p-value < 0.05 and absolute log2FC > 1 for both tests. GO enrichment analysis for these genes is performed using the clusterprofiler package [9], using all genes as background.

To identify differentially spliced genes, we ran ELLIPSIS on the immune cells only using the closest 100 (immune cell) neighbors in the PCA space with the first 20 PCs. Next, we selected genes for which the PSI value was computed for at least 100 microglia-like and 100 macrophage-like cells, to ensure we can pick up gradual effects. For every exon, we computed the correlation of the Ψ-values

with the pseudotime and the difference Δ between the average PSI of the 10 cells that are most microglia-like vs. the cells that are most macrophage-like, using the assigned pseudotime. To obtain a null distribution for Δ, we randomly permute the assigned pseudotimes. We randomly permute the pseudotimes 10 times, and compute Δ for 100 random genes for each permutation. Next, we fit a normal distribution to all the obtained values for Δ. An exon is considered differentially spliced along the trajectory if the observed Δ is not likely to belong to this background distribution (p-value < 0.05, with Benjamini-Hochberg correction for multiple hypothesis testing), and if the correlation of it's Ψ-values with the pseudotime has correlation coefficient > 0.7 or < -0.7 with p-value < 0.05. GO enrichment analysis for the resulting genes is performed using clusterprofiler [9], using only the genes where the Ψ-values are computed in at least 100 microphage-like and 100 microglia-like cells.

7 Optimal parameters for ELLIPSIS

Based on the simulated data with all known transcripts, we performed a grid search to estimate the optimal values for the weights attributed to the conservation of flow equations w_{flow}, the source/sink equations w_{ss}, and the cell-similarity equations w_{sim}. Additionally, we also tested different limits to the maximum number of EM iterations $nIter$. We tested all possible combinations of the following values: $w_{\text{flow}} = [4, 6, 8, 10]$, $w_{\text{ss}} = [1, 3, 5, 10]$, $w_{\text{sim}} = [2, 4, 6, 8]$ and $nIter = [100, 200, 300, 400, 500]$.

After running ELLIPSIS, we computed the weighted average absolute exon error per gene for each parameter combination and looked at the median and mean gene-specific error. The minimum median error (4%) was observed for parameters $nIter = 100$, $w_{\text{ss}} = 1$, $w_{\text{sim}} = 4$, and $w_{\text{flow}} = 6$. While the minimum mean error (6%) was observed for $nIter = 500$, $w_{\text{ss}} = 1$, $w_{\text{sim}} = 6$, and $w_{\text{flow}} = 10$.

To assess the impact of each parameter separately, we considered the optimal parameter combination that minimizes the median exon error: $nIter = 100$, $w_{\text{ss}} = 1$, $w_{\text{sim}} = 4$ and $w_{\text{flow}} = 6$, and changed the value of one parameter at a time.

We observed that the weights for the source/sink equations w_{ss}, had the most impact on the accuracy of the results, although even with $w_{\text{ss}} = 10$, the median error is still limited to 5%, as can be seen in Fig. S10. For Smart-seq based data, the read coverage at the 3' and 5' ends of transcripts is typically decreased because short fragments, resulting from tagmentation near the ends the transcripts, are selected against by Illumina. Therefore, Ψ-values near the end of transcripts are typically slightly underestimated based on the read coverage. However, by heavily imposing that $\Psi_{\text{source}} = \Psi_{\text{sink}} = 1$, we force the exons near the ends of the transcripts to have high Ψ-values, even with low coverage. This in turn results in underestimating the gene expression α, which impacts the accuracy of all exons in the splice graph. By using a smaller weight w_{ss}, we allow a little bit of freedom for the source and sink to account for the lower end-of-transcript coverage.

The values of w_{sim} and w_{flow} have less impact on the overall accuracy of ELLIPSIS, but from Fig S11 and S12 we see that $w_{\text{sim}} = 4$, and $w_{\text{flow}} = 6$ are the optimal values.

Finally, as the majority of genes need less than 100 iterations to finish, increasing the maximum number of iterations does not result in different Ψ-values or longer computation times, see Fig. S13. Therefore, we will keep the default value for the number of iterations to 100. However, when analyzing bigger datasets with large numbers of novel splice events, the expectation-maximization approach might need more iterations to reach convergence. In those cases, it would be advised to use a higher number for $nIter$.

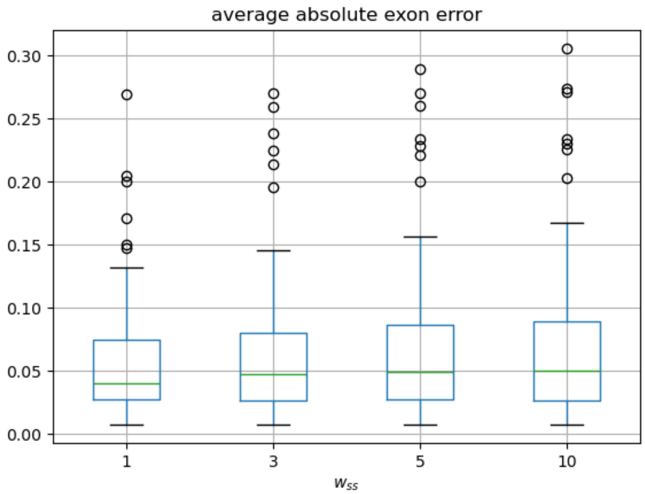

Fig. S10: The distribution of the average absolute exon error per gene for different values of w_{ss}. The other parameters are fixed to $nIter = 100$, $w_{\text{sim}} = 4$ and $w_{\text{flow}} = 6$. The minimum error is reached for $w_{\text{ss}} = 1$

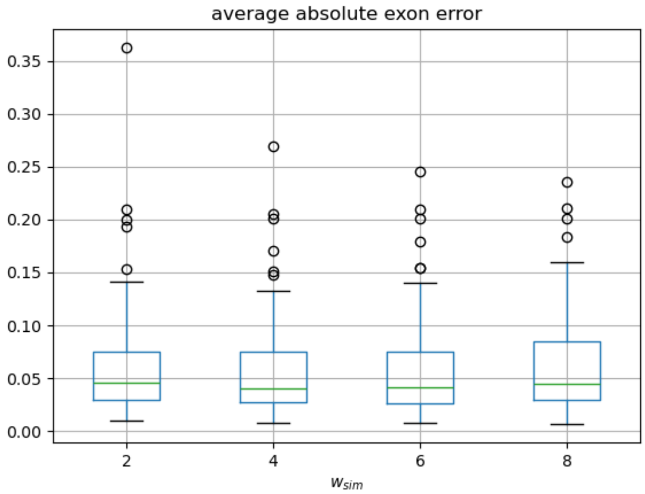

Fig. S11: The distribution of the average absolute exon error per gene for different values of w_{sim}. The other parameters are fixed to $nIter = 100$, $w_{\text{ss}} = 1$ and $w_{\text{flow}} = 6$. The minimum error is reached for $w_{\text{sim}} = 4$

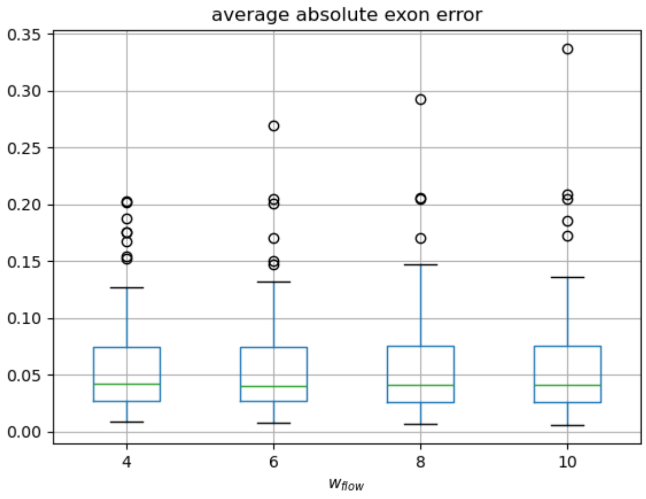

Fig. S12: The distribution of the average absolute exon error per gene for different values of w_{flow}. The other parameters are fixed to $nIter = 100$, $w_{\text{ss}} = 1$ and $w_{\text{sim}} = 4$. The minimum error is reached for $w_{\text{flow}} = 6$

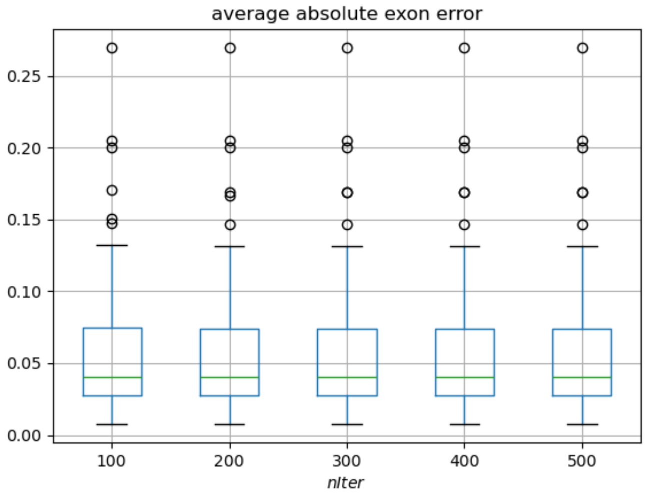

Fig. S13: The distribution of the average absolute exon error per gene for different values of $nIter$. The other parameters are fixed to $w_{\text{ss}} = 1$, $w_{\text{sim}} = 4$ and $w_{\text{flow}} = 6$.

References

[1] Nathan Archer, Mark D Walsh, Vahid Shahrezaei, and Daniel Hebenstreit. Modeling Enzyme Processivity Reveals that RNA-Seq Libraries Are Biased in Characteristic and Correctable Ways. *Cell systems*, 3(5):467–479.e12, nov 2016.

[2] C.F. Buen Abad Najar et al. Identifying cell state-associated alternative splicing events and their coregulation. *Genome research*, 32:1385–1397, 7 2022.

[3] S. Darmanis et al. Single-cell rna-seq analysis of infiltrating neoplastic cells at the migrating front of human glioblastoma. *Cell Reports*, 21:1399–1410, 2017.

[4] J. Gilis et al. saturn: Scalable analysis of differential transcript usage for bulk and single-cell rna-sequencing applications. *F1000Research*, 10:374, 2021.

[5] M. Giulietti et al. Spliceaid-f: a database of human splicing factors and their rna-binding sites. *Nucleic acids research*, 41:D125–D131, 2013.

[6] Y. Hao et al. Dictionary learning for integrative, multimodal and scalable single-cell analysis. *Nature Biotechnology*, 2023.

[7] Y. Huang and G. Sanguinetti. Brie2: computational identification of splicing phenotypes from single-cell transcriptomic experiments. *Genome biology*, 22:251, 8 2021.

[8] Patro R. et al. Salmon provides fast and bias-aware quantification of transcript expression. *Nature Methods*, 14:417–419, 2017.

[9] Tianzhi Wu, Erqiang Hu, Shuangbin Xu, Meijun Chen, Pingfan Guo, Zehan Dai, Tingze Feng, Lang Zhou, Wenli Tang, Li Zhan, Xiaocong Fu, Shanshan Liu, Xiaochen Bo, and Guangchuang Yu. clusterProfiler 4.0: A universal enrichment tool for interpreting omics data. *The Innovation*, 2(3), aug 2021.

[10] W. Zhao et al. Postar3: an updated platform for exploring post-transcriptional regulation coordinated by rna-binding proteins. *Nucleic acids research*, 50:D287–D294, 1 2022.
